# Zygo-Albuside A: New Saponin from *Zygophyllum album* L. with Significant Antioxidant, Anti-Inflammatory and Antiapoptotic Effects against Methotrexate-Induced Testicular Damage

**DOI:** 10.3390/ijms231810799

**Published:** 2022-09-16

**Authors:** Reda F. A. Abdelhameed, Shaimaa A. Fattah, Eman T. Mehanna, Dina M. Hal, Sarah M. Mosaad, Maged S. Abdel-Kader, Amany K. Ibrahim, Safwat A. Ahmed, Jihan M. Badr, Enas E. Eltamany

**Affiliations:** 1 Department of Pharmacognosy, Faculty of Pharmacy, Suez Canal University, Ismailia 41522, Egypt or; 2 Department of Pharmacognosy, Faculty of Pharmacy, Galala University, New Galala 43713, Egypt; 3 Department of Biochemistry, Faculty of Pharmacy, Suez Canal University, Ismailia 41522, Egypt; 4 Division of Pharmacology and Therapeutics, General Authority of Healthcare, Ismailia 41522, Egypt; 5 Department of Pharmacognosy, College of Pharmacy, Prince Sattam Bin Abdulaziz University, Al-Kharj 11942, Saudi Arabia; 6 Department of Pharmacognosy, Faculty of Pharmacy, Alexandria University, Alexandria 21215, Egypt

**Keywords:** *Zygophyllum album* L., saponins, antioxidant activity, antiapoptotic, testicular damage

## Abstract

Chemical investigation of the crude extract of the aerial part of *Zygophyllum album* L. (*Z. album*) led to the isolation of a new saponin, Zygo-albuside **A** (**7**), together with seven known compounds, one of them (caffeic acid, compound **4**) is reported in the genus for the first time. NMR (1D and 2D) and mass spectrometric analysis, including high-resolution mass spectrometry (HRMS), were utilized to set up the chemical structures of these compounds. The present biological study aimed to investigate the protective antioxidant, anti-inflammatory, and antiapoptotic activities of the crude extract from the aerial part of *Z. album* and two of its isolated compounds, rutin and the new saponin zygo-albuside **A**, against methotrexate (MTX)-induced testicular injury, considering the role of miRNA-29a. In all groups except for the normal control group, which received a mixture of distilled water and DMSO (2:1) as vehicle orally every day for ten days, testicular damage was induced on the fifth day by intraperitoneal administration of MTX at a single dose of 20 mg/kg. Histopathological examination showed that pre-treatment with the crude extract of *Z. album*, zygo-albuside **A**, or rutin reversed the testicular damage induced by MTX. In addition, biochemical analysis in the protected groups showed a decrease in malondialdehyde (MDA), interleukin-6 (IL-6) and IL-1β, Bcl-2-associated-protein (Bax), and an increase in B-cell lymphoma 2 (Bcl-2) protein, catalase (CAT), superoxide dismutase (SOD) in the testis, along with an increase in serum testosterone levels compared with the unprotected (positive control) group. The mRNA expression levels of nuclear factor-kappa B (NF-κB), tumor necrosis factor-α (TNF-α), p53, and miRNA-29a were downregulated in the testicular tissues of the protected groups compared with the unprotected group. In conclusion, the study provides sufficient evidence that *Z. album* extract, and its isolated compounds, zygo-albuside **A** and rutin, could alleviate testicular damage caused by the chemotherapeutic agent MTX.

## 1. Introduction

Methotrexate (MTX) is a folate antagonist that has been successfully used to treat various types of malignant tumors as well as autoimmune diseases [1]. One of the main obvious side effects of MTX cytotoxicity is damage to the testis, leading to sterility [2]. The latter has been associated with oxidative stress, inflammatory cytokines, and apoptotic cascades [3,4]. A close relationship has been established between MTX and oxidative stress, leading not only to an increase in reactive oxygen species (ROS) but also to a decrease in antioxidant defense mechanisms such as antioxidant enzymes such as catalase (CAT) and superoxide dismutase (SOD) and non-enzymatic antioxidants such as reduced glutathione (GSH) [5,6,7]. Furthermore, inflammatory reactions in the male genital system are closely related to oxidative stress [8]. The pathogenesis of MTX-induced testicular damage is also associated with the production of proinflammatory cytokines such as interleukin-6 (IL-6) and IL-1β and cell death via apoptotic cascades [3,6]. However, the exact processes that cause MTX-induced testicular damage are still unknown.

MicroRNAs (miRNAs) are short, non-coding RNAs of about 22 nucleotides that have been associated with the control of various physiological processes such as cell proliferation, cell death, fibrosis, inflammation, and metabolism [9,10,11]. The family of miRNA-29s consists of three members; miRNA-29a, miRNA-29b, and miRNA-29c. miRNA-29a is an important member of the miRNA-29 family and has been shown to exhibit proapoptotic activity [11,12]. Recently, administration of MTX showed upregulation of testicular miRNA-29a expression, which is associated with apoptosis, oxidative stress, and inflammation [13].

Several studies have proved that phytotherapeutic antioxidants are considered as an auspicious strategy for attenuating chemotherapy-induced organ toxicities [14,15,16,17,18,19,20,21,22,23]. Additionally, numerous natural products have been reported for being beneficial in the management of male reproductive problems [24] arising from environmental pollutants, toxins and drugs [25]. Various plant extracts and natural products have been evidenced to boost testosterone, improve the sperm quality, positively influence the androgen status, semen parameters and fertility index [26,27,28,29,30] as well as counteracting the gonadotoxicity induced by antineoplastic agents [13,24,25,31,32,33], D-galactose [34,35], heat [36,37] and radiation [38]. Such protective effects of the extracts are attributed to their antioxidant and anti-inflammatory components [13,24,25,31,32,33,34,35,36,37,38], most predominantly saponins [25,35,36,37]. Saponins are the most famous testosterone boosters that enhance testosterone production or function as antiestrogenic agents via aromatase or estrogen receptors inhibition [30]. They are responsible for the androgenic effect of *Tribulus terrestris* (Zygophyllaceae) [28]. Furthermore, date palm pollen saponins increased the testosterone production via the stimulation of Leydig cells of the testis [29]. The beneficial effects of ginseng on the male reproductive system are attributed to its saponin components known as ginsenosides [39]. Moreover, steroidal saponins of *Dioscorea* Genus (Yam) are used in the manufacture of steroidal hormones including sex hormones [40].

Zygophyllaceae (Caltrop family) comprises about 25 genera and 240 species among which six genera viz. *Tetradiclis, Seetzenia,* *Fagonia*, *Peganum*, *Tribulus* and *Zygophyllum album* (*Z. album*) are widespread in Egypt [41]. The genus *Z. album* is represented in Egypt by nine species [42,43], which are characterized by being succulent plants that resist drought and salt. These characters enable them to survive under drought, dry climatic situations [44]. Plants of genus *Zygophyllum* are employed by traditional healers in Egypt for the treatment of several ailments such as asthma, hypertension, diabetes, gout, rheumatism, and dysmenorrhea. Moreover, several biological studies evidenced their diverse pharmacological activities [43]. For instance, *Z. album* was reported to possess promising antioxidant [45,46,47,48], antidiabetic [46,47,49,50,51], antihyperlipidemic [47,49,52], anti-obesity [52], anti-acetylcholinesterase [52], antihypertensive [50], anticancer [47], cardioprotective and anti-inflammatory activities [53]. *Z. album* owes such pharmacological effects to an assortment of phytochemicals. Chemical investigation of *Z. album* revealed that the plant contains volatile oil [54] and accumulates triterpenes [43], saponins [43,55,56,57,58], sterols [43,55], phenolic compounds predominantly flavonoids [43,47,52,57,59], as well as few acids and their derivatives [52].

Based on the aforesaid considerations, besides that the impact of *Z. album* versus MTX-induced testicular injury has not been explored yet, we continued our efforts in discovering bioactive compounds from Egyptian folk medicine. Therefore, we investigated the chemical constituents of *Z. album* aerial parts and then studied the potential protective effects of *Z. album* crude extract together with two of the isolated pure compounds against methotrexate-induced testicular damage.

## 2. Results

### 2.1. Structure Elucidation of the Isolated Compounds

**Figure 1:**
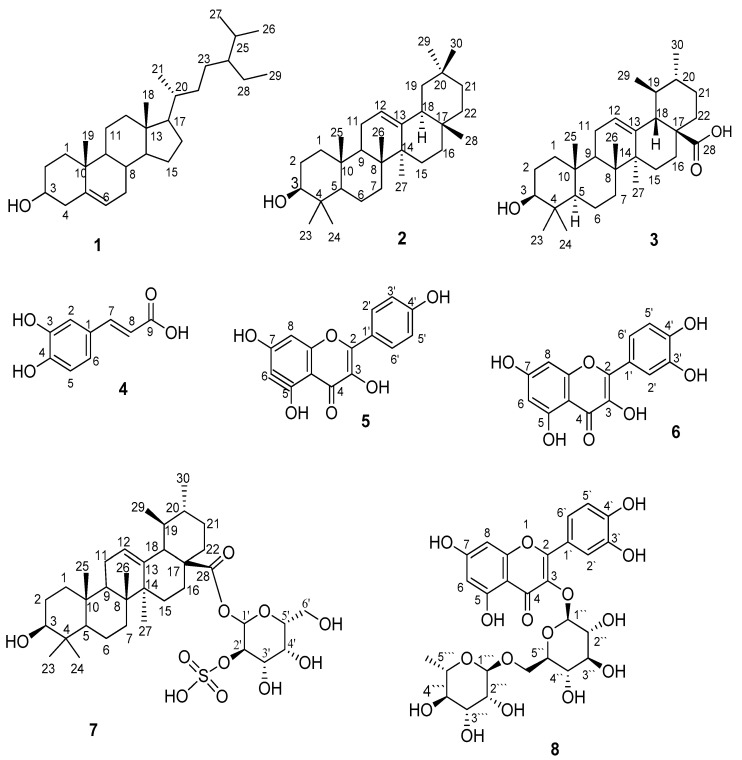
Chemical structures of the isolated compounds.

The chemical structures of compounds **1**–**6** and **8** were identified based on co-chromatography with the available authentic samples and were further confirmed by comparing their ^1^H NMR and ^13^C NMR spectra data (Figures S1–S12, S30 and S31) with those previously reported in the literature. The chemical structures of the isolated compounds are displayed in Figure 1. Compound **1** was identified as *β*-sitosterol [60]. Compounds **2** and **3** were found to be triterpenes identified as *β*-amyrin and ursolic acid, respectively [61,62,63]. Compound **4** was characterized as caffeic acid [64]. Compounds **5**, **6**, and **8** were interpreted as flavonoids, namely kaempferol [65], quercetin [65,66], and rutin [67], respectively.

**Figure 2:**
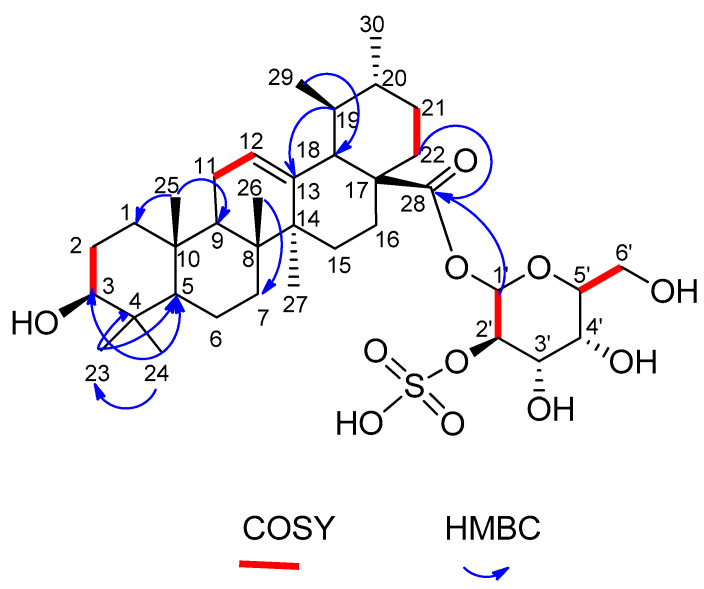
Selected COSY and HMBC correlations of compound **7**.

Compound **7** (Figure 1 and Figure 2) was isolated as a white powder with a molecular formula of C_36_H_58_O_11_S as deduced from its ^1^H and ^13^C NMR spectral data (Table 1) and further confirmed by 2D NMR analysis and HRMS, which displayed a molecular ion peak at *m/z* 697.2885 [M-H]^−^ (Figure S13). The positive results of rhodizonate test indicated the existence of a sulfate group [68,69,70,71].

Extensive inspection of ^1^H NMR and ^13^C NMR data (Figures S14–S21) in addition to HSQC (Figures S27–S29) allowed for the assignment of five tertiary methyls detected at δ_H/C_ 0.90/17.5, 1.03/28.4, 1.00/19.9, 0.93/19.9 and 1.10/13.6 assignable for H_3_/C-25, 23, 26, 24 and 27, respectively. This assignment was confirmed from HMBC correlations. For example, the signal detected a δ_H_ 0.93 assignable for H_3_-24 was correlated to the signal resonated at δ_C_ 28.4, 55.6 and 74.9 assignable for C-23, C-5 and C-3, respectively. The signal detected at δ_H_ 1.03 (for H_3_-23) was correlated to δ_C_ 38.9 and 55.6 assignable for C4 and C-5. In addition, the singlet at δ_H_ 0.90 (H_3_-25) was linked in HMBC spectrum (Figures S25 and S26) to each of δ_C_ 39.0 and 47.1 assignable for C-1 and C-9, respectively. Similarly, other selected correlations are indicated in Figure 2.

The two methyls displayed at δ_H/C_ 0.94 (d, J = 8.0 Hz)/21.4 and 1.11 (d, J = 10.0 Hz)/17.6 were attributed to H_3_-30/C-30 and H_3_-29/C-29, respectively. Additionally, the oxygenated methine proton detected at δ_H_ 3.64 (dd, J = 5, 10 Hz) together with its corresponding carbon detected at δ_C_ 74.9 were assignable to H-3/C-3. The location of the hydroxyl group was further confirmed from the correlation between the singlet resonating at δ_H_ 0.93 (for H_3_-24) and the carbon signal detected at δ_C_ 74.9 (for C-3). Moreover, H-3 (δ_H_ 3.64) was linked to H_2_-2 (δ_H_ 1.25 and 1.42) as shown from the COSY spectrum (Figures S23 and S24).

An olefin proton was detected at δ_H_ = 5.35 (m) attributed to H-12 with its corresponding carbon at δ_C_ 126.1 (C-12). Another sp^2^ carbon resonated at δ_C_138.5, and this confirmed the classical double bond between C-12 and C-13. Another confirmation for the situation of the double bond was obtained from the COSY spectrum that revealed the link between H_2_-11 (δ_H_ 1.96 and 2.15) and H-12 (δ_H_ 5.35). Additionally, HMBC spectrum illustrated correlation between C-13 (δ_C_ 138.5) and H-19 (δ_H_ 1.11).

The ^13^C NMR spectrum displayed resonances for characteristic signal at δ_C_ 172.6 for a carbonyl functionality of an ester; this signal disclosed a long range coupling with H_2_-22 (δ_H_ 1.41 and 2.06) as exhibited by HMBC spectrum.

The DEPT-135 (Figure S22) allowed differentiation of the 36 carbon resonances into 7 methyl, 9 methylene, 7 methine, and 7 quaternary carbons attributed to the aglycon moiety. These data indicated an ursane-type triterpene skeleton for the aglycon with one carboxy group at C-28 [57,58,72]. The remaining six carbon signals were assigned to the sugar moiety which was identified as galactose. ^13^C NMR spectra displayed the characteristic signal of an anomeric carbon at *δ*_C_ 86.8, which appeared shielded due to the presence of the -SO_3_H group at C-2′. The anomeric carbon for galactose usually appears around 99.2 [73], but in the presence of the -SO_3_H group at C-2′, it moves up the field to 86.8 [72]. The remaining signals at *δ*_C_ 78.5, 73.3, 68.9, 74.6, and 60.3 indicate the presence of a sugar part.

The anomeric proton exhibited a coupling constant at *δ*_H_ 4.75 (1H, d, *J* = 10.0 Hz, H-1′), which is characteristic to the di-axial interaction between H-1′ and H-2′; this value, besides the chemical shift of the anomeric carbon at *δ*_C_ 86.8, assured the *β*-configuration [74,75,76].

The situation of the sugar moiety was indicated from the long-range coupling between the anomeric proton resonating at δH 4.75 and the carbonyl functionality detected at δC 172.6 as revealed from the HMBC spectrum. To place emphasis on the structure of compound **7**, it was subjected to acid hydrolysis [77] followed by co-chromatography of the resulted aglycone and sugar parts of compound **7** with authentic triterpenes and sugars, respectively. The aglycone part was assured to be ursolic acid (compound **3**) (R_f_ = 0.3, 0.64 and 0.71 mobile phase: benzene/toluene (1:4), toluene/EtOAc/AcOH (6:3:1) and 3% MeOH in CHCl_3_ [78,79,80]. The sugar moiety was authenticated to be galactose (Rf = 0.21, 0.38 and 0.39; mobile phase: *n*-butanol-benzene-pyridine-HO (5:1:3:3), PhOH satd. with H_2_O and propanol-H_2_O (8.5:1.5), respectively) [81,82].

Based on the above including the ^1^H and ^13^C NMR data, which were compared with those previously published for Zygophyllum saponins [57,58,83] and confirmed by HMBC and COSY correlations (Figure 2), compound (**7**) was elucidated as a new compound isolated for the first time from a natural source and given the name Zygo-albuside **A.**

### 2.2. In Vitro Antioxidant Assessment of Compound ***7*** (Zygo-Albuside **A**)

Plant antioxidants frame a wide assortment of phytochemicals that exert their antioxidant action by various mechanisms such as including quenching of singlet oxygen, hydrogen convey, transport of electron in addition to reduction of metal and chelation [84,85]. Previous studies have proven the antioxidant activity of *Z. album* different extracts and fractions [46,48,86,87]. This encouraged the inspection of the antioxidant efficacy of compound **7** (Zygo-albuside **A**) in the current study by utilizing three indicative assays (DPPH, H_2_O_2_, TAC).

The DPPH radical has been frequently utilized to explore the neutralizing effects of crude extracts or pure compounds. Antioxidants can pause the free radicals’ chain of oxidation reactions via creating more stable radicals. Thus, they can scavenge the DPPH radical through donation of hydrogen, giving rise to transformation of the purple-colored DPPH radical into the yellow-colored DPPH-H [88,89].

In this study, the results depicted in Table 2 revealed that compound **7** possessed promising radical scavenging ability with IC_50_ = 45.41 ± 2.65 µg/mL compared to ascorbic acid as positive control (IC_50_ = 10.64 ± 0.82 µg/mL).

Furthermore, hydrogen peroxide (H_2_O_2_) is a powerful oxidant, playing a decisive role in cell signaling pathways [89]. In biological systems, H_2_O_2_ is produced by a number of oxidizing enzymes such as SOD [90]. However, the accumulation of H_2_O_2_ can cause oxidative stress and inflammatory reactions [89,91,92]. Decomposition of H_2_O_2_ into the hydroxyl radical (•OH) initiates lipid peroxidation and causes cellular components injury, giving rise to several inflammatory conditions [93]. Accordingly, controlling H_2_O_2_ production by natural antioxidants could be of great benefit [89]. Compound **7** exhibited good H_2_O_2_ quenching effect with an IC_50_ = 65.16 ± 3.22 (Table 2).

The in vitro total antioxidant capacity (TAC) of compound **7** was assessed by application of phosphomolybdate assay, which depends on the generation of a green phosphate/MoV complex. This complex is created due to the reduction of phosphomolybdate ion by the compound with an antioxidant effect [84]. Results were expressed as milligram of gallic acid equivalent per gram of dry extract (mg GAE/g).

Accordingly, compound **7** exhibited noteworthy TAC (29.83 ± 2.19 mg GAE/g) matched with ascorbic acid (the positive control; 71.28 ± 4.34 mg GAE/g) pointing to its potent capacity to remove free radicals by an electron transfer mechanism (Table 2).

### 2.3. In Vivo Evaluation of Z. album Extract, Compound ***7*** (Zygo-Albuside **A**) and Compound ***8*** (Rutin)

#### 2.3.1. Effect on Liver and Kidney Function Markers (Preliminary Study)

In order to assess the possible toxicity of the administered compounds on the liver and kidney, a preliminary study was conducted to determine the serum levels of the liver enzymes alanine aminotransferase (ALT), and aspartate aminotransferase (AST), and the kidney markers urea and creatinine in mice that received only *Z. album* extract (100 mg/kg), zygo-albuside **A** (10 mg/kg) and rutin (10 mg/kg). No significant differences were observed in the levels of both liver and kidney function markers in any of the treated groups relative to the negative control mice (Table S1), indicating that the investigated doses had no detected toxicity on either the liver or the kidney (Table S1). No other toxic effects were detected in the experimental mice. There were also no observed changes in the behavior of the treated mice nor a marked increase in their mortality rates.

#### 2.3.2. Reversing Serum Testosterone Levels in the MTX-Administrated Mice

**Figure 3:**
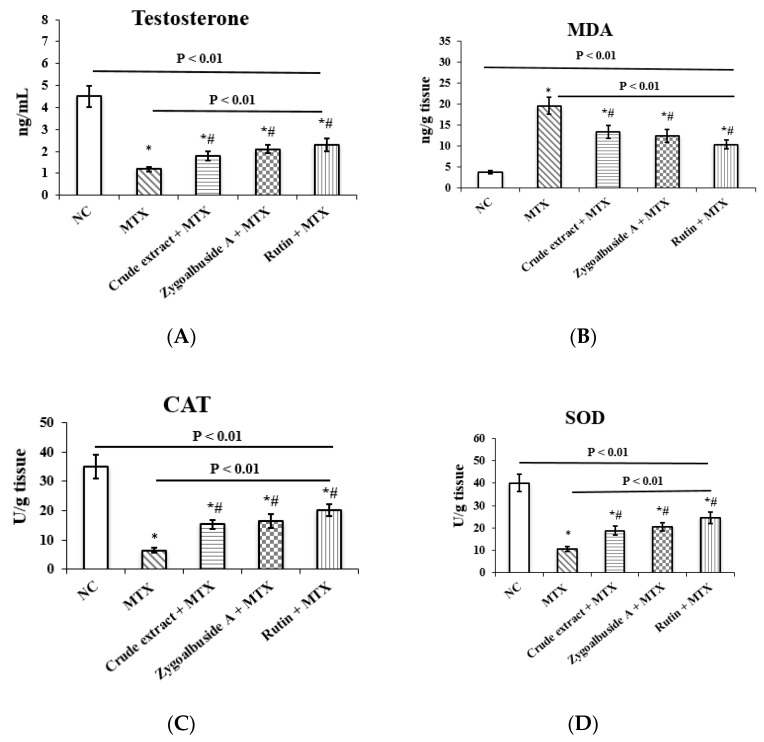
Effect of administration of *Z. album* crude extract, zygo-albuside **A** and rutin on serum levels of (**A**) testosterone, and testicular levels of (**B**) MDA, (**C**) CAT and (**D**) SOD in MTX-induced testicular injury mice. Statistical analysis was performed using ANOVA followed by Tukey’s post hoc test. Values are expressed as mean ± SD (n = 8). A statistically significant difference was assumed at *p* < 0.01 and denoted by * vs. NC; # vs. MTX. MDA; malondialdehyde, CAT; catalase, SOD; superoxide dismutase.

The results in Figure 3A show that administration of MTX in the principle investigative study was associated with a significant 73% decrease in serum testosterone levels compared with the normal control (NC) group. MTX-injected mice pretreated with either the crude extract of *Z. album*, zygo-albuside **A**, or rutin showed a significant increase in serum testosterone levels by 33%, 42.8%, and 47.8% compared to the untreated MTX (positive control) group.

#### 2.3.3. Restoring the Antioxidant Activity in the MTX-Administrated Mice

As shown in Figure 3B, testicular malondialdehyde (MDA) levels were significantly increased by 80.6% in the MTX group compared with the NC group. Co-administration of *Z. album* crude extract, zygo-albuside **A**, or rutin with MTX significantly decreased testicular tissue MDA levels by 31.6%, 36.7%, and 46.9%, respectively, compared with the MTX positive control group.

Compared with NC, a significant decrease in the activities of CAT and SOD (73.5% and 81.4%, respectively) was observed after exposure to MTX. Concurrent treatment with *Z. album* crude extract, zygo-albuside **A**, or rutin showed significant restoration of the levels of these testicular antioxidants compared with mice injected with MTX alone (Figure 3C,D).

#### 2.3.4. Reduction of Inflammation in the MTX-Administrated Mice

**Figure 4:**
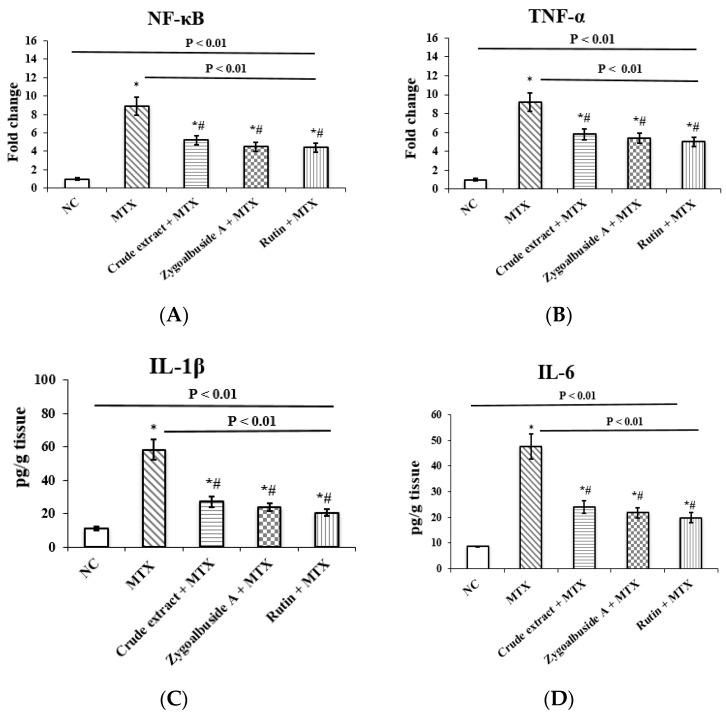
Testicular gene expression of (**A**) NF-κB, (**B**) TNF-α and tissue levels of (**C)** IL1-β and (**D**) IL-6 in the normal control (NC) group and experimental groups (MTX, crude extract + MTX, zygo-albuside **A** + MTX, rutin + MTX). Statistical analysis was performed using ANOVA followed by Tukey’s post hoc test. Values are expressed as mean ± SD (n = 8). A statistically significant difference was assumed at *p* < 0.01 and denoted by * vs. NC; # vs. MTX. NF-κB; nuclear factor kappa B, TNF-α; tumor necrosis factor-α, IL-1β; interleukin-1β, IL-6; interleukin-6.

MTX administration was associated with significant inflammatory changes in testicular tissue, as indicated by significant increases in the expression levels of nuclear factor-kappa B (NF-κB), tumor necrosis factor-α (TNF-α) by 8.9- and 9.2-fold, respectively, and a significant increase in IL-1β and IL-6 levels by 80.7%, and 81.7%, respectively, compared with the NC group (Figure 4A–D).

MTX-injected mice pretreated with either *Z. album* crude extract, zygo-albuside **A**, or rutin showed significant decreases in NF-κB expression levels by 5.2-, 4.5-, and 4.4-fold, respectively, and in TNF-α expression levels by 5.8-, 5.4-, and 5-fold, respectively, compared with the unprotected (positive control) group. Apparently, the isolated compounds also had a significant effect on IL-1β and IL-6 levels by decreasing them compared to the MTX positive control group. *Z. album* crude extract, zygo-albuside **A**, or rutin exhibited a significant decrease in IL-1β levels by 53.3%, 59.1%, and 64.6%, respectively, and in IL-6 levels by 49.3%, 53.9%, and 58.4%, respectively (Figure 4A–D).

#### 2.3.5. Prevention of Apoptosis in the MTX-Administrated Mice

**Figure 5:**
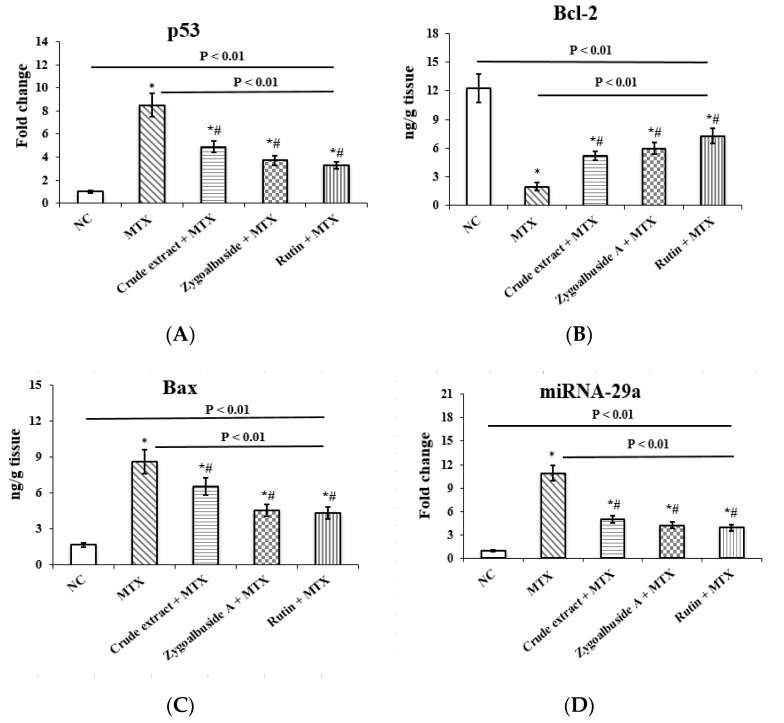
Effect of administration of crude extract, zygo-albuside **A**, or rutin on testicular expression of (**A**) p53, (**B**) miRNA-29a, mRNA and testicular levels of (**C**) Bax and (**D**) Bcl-2 in mice with MTX-induced testicular injury. Statistical analysis was performed using ANOVA followed by Tukey’s post hoc test. Values are expressed as mean ± SD (n = 8). A statistically significant difference was assumed at *p* < 0.01 and denoted by * vs. NC; # vs. MTX.

In response to cellular stress, the tumor suppressor p53 leads to cell cycle arrest and apoptosis [94]. In this context, administration of MTX resulted in a significant 8.5-fold increase in p53 expression in the testis compared to the NC group. This effect was attenuated by pre-administration of *Z. album* crude extract, zygo-albuside **A**, or rutin by 4.9-, 3.7-, and 3.3-fold, respectively, compared to the MTX positive control group (Figure 5A).

Members of the Bcl-2 family play an important role in testicular cell survival by regulating both caspase-dependent and caspase-independent cell death. Bcl-2 is an antiapoptotic protein belonging to the family of apoptosis-regulating proteins [95]. The current results showed that only MTX-injected mice had 83.9% lower Bcl-2 levels than the NC group. Mice pretreated with *Z. album* crude extract, zygo-albuside **A** or rutin had 62%, 67% and 72% higher Bcl-2 levels, respectively, compared to the MTX positive control group (Figure 5B).

Furthermore, Bax levels were significantly increased by 81% in mice administered MTX only compared with the NC group. Pretreatment with *Z. album* crude extract reduced Bax levels by 24% compared with untreated mice. Stronger effects were apparently caused by zygo-albuside **A** or rutin, which reduced Bax levels by 47% and 45%, respectively, compared with the MTX positive control group (Figure 5C).

#### 2.3.6. Inhibition of mi-RNA 29a Expression in MTX-Administrated Mice

The expression of mi-RNA 29a was significantly increased by 10.9-fold in MTX-injected mice relative to the NC group. Oral administration of *Z. album* crude extract, Zygo-albuside **A**, or rutin significantly decreased mi-RNA 29a expression by 54%, 61%, and 64%, respectively, compared with the MTX positive control group (Figure 5D).

#### 2.3.7. Improvement of the Histological Damage in the Testis

**Figure 6:**
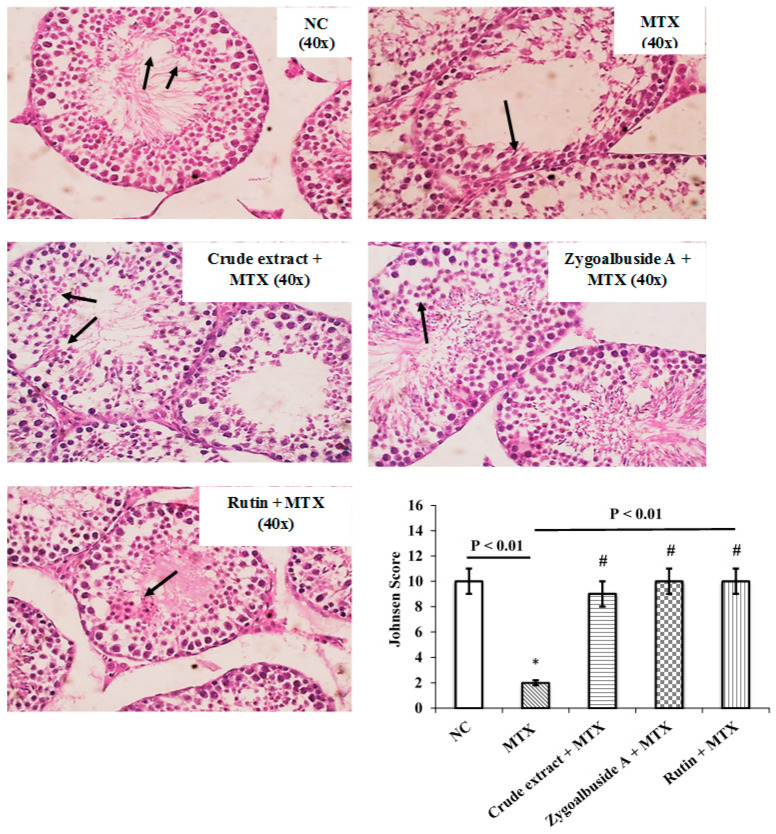
Representative photomicrographs of testis from control and experimentally treated mice. Testicular tissue sections stained with hematoxylin and eosin (40×) and scored by Johnsen. The normal control group (NC) showed complete spermatogenesis (black arrow) in all tubules, up to the sperm count (score 10). In the MTX group, some tubules are hyalinized, while other tubules show a marked reduction in spermatogenesis with few spermatocytes (black arrows) (score 2). The Crude extract + MTX group showed tubules with complete spermatogenesis (black arrows) and slightly impaired spermatogenic cells (score 9). The Zygo-albuside **A** + MTX group showed the most pronounced effect; complete spermatogenesis is evident in all tubules up to spermatids (black arrow) (score 10). The Rutin + MTX group also showed complete spermatogenesis in all tubules, up to the spermatids (black arrow) (score 10). Scores are expressed as mean ± SD (n = 8). Statistical analysis was performed by one-way analysis ANOVA followed by Tukey’s post hoc test. A statistically significant difference was assumed at *p* < 0.01 and marked with * vs. NC; # vs. MTX.

As shown in Figure 6, a normal structure was observed in the testis of the control mice, and the mice receiving zygo-albuside **A** and rutin exhibited normal architecture of the testicular tubules with complete spermatogenesis. In contrast, the group injected with MTX (positive control group) showed hyalinized tubules with a significant decrease in spermatogenesis compared to the NC group. In addition, a significant decrease in Johnsen score in the group injected with MTX was observed compared to the normal testicular parts. Treatment with *Z. album* crude extract improved the Johnsen score nine-fold compared with the unprotected group. Maximum protection was achieved by zygo-albuside **A** and rutin compounds, which brought the score to normal.

## 3. Discussion

Chemotherapeutic drugs have always been associated with persistent azoospermia and infertility in men. MTX can damage testicular tubules, reduce sperm count, and cause genetic abnormalities [96]. Consistent with previous studies, the present study found that a single i.p. dose of 20 mg/kg MTX can cause testicular damage. In this context, great attention should be paid to these side effects, and new natural products should be sought to counteract MTX-induced testicular damage.

Our histological findings showed damage to testicular tissue with hyalinized testicular tubules and a significant decrease in the Johnsen score for spermatogenesis after injection of MTX compared with NC. Consequently, MTX resulted in a significant decrease in testosterone levels compared with NC. Several research papers supported our findings demonstrating the reproductive toxicity of MTX [13,34,96].

It is reported that the toxicity of MTX is triggered by several mechanisms, including oxidative stress, inflammation, and apoptosis [35,55,97]. Exposure to X-rays, toxins, and chemicals in the environment, as well as certain physical disorders such as varicocele, can increase oxidative stress and promote germ cell death and thus spermatogenesis [98]. Sperm abnormalities and infertility are caused by uncontrolled ROS production and oxygen-induced lipid peroxidation of the abundant polyunsaturated fatty acids in the sperm membrane [99,100]. In this regard, tissue levels of MDA, a marker of lipid peroxidation [5], were increased in the MTX group compared to NC. In addition, the MTX group showed a significant decrease in testicular levels of the antioxidant enzymes SOD and CAT compared to NC; similar results were previously observed [13,96,97]. The body’s ability to produce antioxidants under normal circumstances to counteract the negative effects of oxidative stress is influenced by metabolic processes and genetic structure. In addition, environmental variables such as diet, pollution, and chemicals can affect this ability. As a result, the body’s antioxidant system cannot neutralize all free radicals and prevent the harmful effects of oxidative stress alone [98].

Furthermore, previous studies have shown that MTX- induced testicular injury is associated with a marked increase in pro-inflammatory cytokines [3,6,101,102]. An increase in TNF-α and/or IL-1β following testicular injury activates the JNK pathway, a stress-related kinase that plays an important role in inflammatory diseases [103] and controls the maturation and function of T cells as well as the production of ROS and pro-inflammatory cytokines such as IL-6 and TNF-α [101]. Moreover, binding of TNF to TNFR1 induces degradation of IκBα, triggering the canonical NF-κB signaling mechanism that activates proinflammatory cytokine genes such as IL-6 and IL-1β [104]. In this regard, previous studies have shown that NF-κB can be stimulated by TNF-α in Sertoli cells [101]. In agreement with previous studies, our results have shown that injection of MTX led to a significant increase in testicular levels of IL-6 and IL-1β, which may be reflected by the upregulation of NF-κB and an increase in TNF-α expression in the testis, compared with NC.

Apoptosis has also been implicated in the pathophysiology of MTX-induced gonadotoxicity [4]. Apoptosis is triggered by either the receptor-mediated extrinsic pathway or the mitochondria-dependent intrinsic mechanism. The extrinsic pathway is activated when extracellular apoptosis-inducing stimuli such as TNF-α dock to death receptors and activate the initiator caspase 8. Members of the Bcl-2 family with anti- or pro-apoptotic activities, such as Bcl-2 and Bax, control the mitochondria-dependent intrinsic pathway. Bcl-2 can prevent cell death by preserving the integrity of the mitochondrial membrane and blocking cytochrome c release. Bax furthermore translocates into mitochondria and promotes the release of cytochrome c, eventually leading to the activation of initiator caspase 9. Both initiator caspases can then activate executioner caspase 3, leading to apoptotic cell death [105]. Sheikhbahaei et al. [4] found that DNA damage induced after injection of MTX activates the p53 gene, which in turn enhances Bax expression. Consistent with the previous studies [4,13,106], the present study demonstrated significantly decreased Bcl-2 levels in MTX-treated mice compared with the NC group. In addition, the expression of p53 and Bax levels were significantly increased in the testicular tissues of the MTX-group compared with the NC group.

Upregulation of miRNA-29 family expression, particularly miRNA-29a, was reported in testis by injection of estradiol benzoate and doxorubicin, both of which promote germ cell apoptosis [107]. Members of the miRNA-29 family have been shown to induce apoptosis in a p53-dependent manner [108]. The present study agrees with the study by Sherif et al. [13] in which the group treated with MTX showed significant miRNA-29a expression compared to NC. Other previous studies have identified a molecular pathway linking miRNA-29a overexpression to oxidative stress activation via increased MDA and decreased SOD levels [12]. Moreover, overexpression of miR-29a increased the production of IL-6 and IL-1 as well as NF-*κ*B activation [109].

Considering that oxidative stress, inflammation, and apoptosis caused by MTX are serious problems that lead to organ failure and have been shown to damage the male reproductive system, the present study aimed to investigate whether oral treatment with *Z. album* extract and two of its isolated compounds can alleviate testicular damage induced by MTX in mice. We investigated a new natural product isolated from *Z. album*, zygo-albuside **A**, which showed promising protective effects against MTX-induced testicular damage. In addition, various protective effects of *Z. album* crude extract or rutin against testicular damage were observed. In the groups injected with MTX and pretreated with *Z. album* crude extract, zygo-albuside **A**, or rutin, histopathological examination showed complete spermatogenesis in all tubules compared to the positive control MTX-injected mice.

The present study revealed the structural characterization of the new compound zygo-albuside **A** obtained from *Z. album*. Zygo-albuside **A** is a saponin consisting of a sugar moiety glycosidically linked to a hydrophobic aglycone. Saponin is one of the most popular structures among natural products used in traditional medicine against various disease mechanisms. In this context, saponin from *Agave brittoniana* Trel subspecies brachypus showed anti-inflammatory activity [110]. Moreover, a previous study elucidated the anti-inflammatory effect of saponins from the roots of *Impatiens parviflora* DC [111]. In the present study, histopathological improvement of testicular tissue and increases in testosterone levels were associated with significant downregulation of NF-κB and TNF-α mRNA and decreases in testicular levels of IL-1β and IL-6 in the zygo-albuside **A**-treated group. Similar histopathological improvements and anti-inflammatory effects were observed in the groups treated with the crude extract of *Z. album* and rutin. The anti-inflammatory properties of *Z. album* have been previously described as cardioprotective, and hepatoprotective [55] effects were previously reported, mainly through suppressing the NF-κB pathway. Moreover, rutin was postulated to exert anti-inflammatory effects via inhibition of the NF-κB signaling pathway [112]. Based on the current results, we propose that the *Z. album* crude extract, zygo-albuside **A**, and rutin may have anti-inflammatory effects by suppressing the activation of NF-κB along with the pro-inflammatory cytokines IL-1β, IL-6, and TNF-α.

Saponins that protect against oxidative stress have received increased attention. It is known from the literature that Notoginsenoside R1 may be able to repair cell damage in neurons by suppressing ROS [113]. Moreover, ginsenoside Rg1 protected human neuroblastoma cells from hydrogen peroxide-induced damage [114]. Consistent with these findings, the present study showed that the saponin zygo-albuside **A** reduced MTX-induced testicular damage by restoring normal levels of testicular antioxidant enzymes such as SOD and CAT and by decreasing testicular MDA levels compared with the MTX positive control group. The same results were obtained for rutin and the crude extract of *Z. album*. These results agree with previous reports on *Z. album* [55]. This could be due to its flavonoid content. Vardi et al. [5] reported the effect of flavonoids on lowering serum MDA and NO and increasing SOD and CAT. Rutin specifically was reported to protect against testicular ischemia–reperfusion-induced oxidative stress in rats through decreasing MDA and increasing SOD and CAT activities [115]. The study by Alsharif et al. [116] reported that inhibition of the action of TNF-α ameliorated dexamethasone-induced oxidative stress in mice. Therefore, we hypothesize a relationship between the anti-inflammatory effect of *Z. album* extracts and their antioxidant capacity.

Furthermore, ginsenoside Rb1 showed an anti-apoptotic effect in Rattus pancreatic β-cells via decreasing caspase-3 gene expression [117]. In addition, the high flavonoid content of some natural products contributes to their anti MTX effect by suppressing various apoptotic pathways and DNA damage [4,5,34]. In the current study, the zygo-albuside **A** saponin as well as the rutin flavonoid showed anti-apoptotic effect by significantly reducing p53 mRNA expression in the testis with concomitant reduction in Bax and an increase in Bcl-2 levels. More to the point, a previous study showed that TNF-α and IL-6 levels are inversely correlated with Bcl-2 expression [118]. Moreover, antioxidants inhibit apoptosis by downregulating p53 and caspase enzymes [119]. Consequently, the current results suggest that the anti-inflammatory effect of the isolated compounds of *Z. album* is not only related to their antioxidant activity, but also to their anti-apoptotic effect in the treatment of testicular damage caused by MTX.

Several natural products have been shown to be highly effective drugs for treatment of a variety of clinical conditions through their influence on miRNA expression [120,121]. Consistent with previous findings, oral administration of the crude extract of *Z. album*, the new natural product zygoalbusid A, or rutin attenuated testicular damage caused by injection of MTX by downregulating testicular miRNA-29a whose anti-inflammatory, antioxidant, and anti-apoptotic effects have been demonstrated in previous studies [99,122,123].

**Figure 7:**
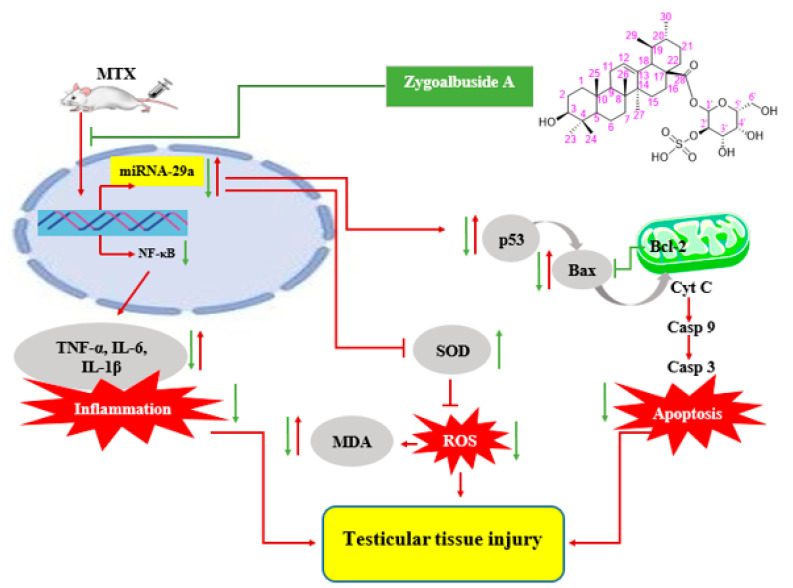
Protective mechanism of zygo-albuside **A** against methotrexate-induced testicular damage. Red arrows indicate the effects of MTX injection. Green arrows indicate the effects of zygo-albuside **A** administration. MTX; methotrexate, NF-κB; nuclear factor kappa B, TNF- α; tumor necrosis factor-α, IL-1β; interleukin 1β, IL-6; interleukin-6, SOD; superoxide dismutase, ROS; reactive oxygen species, MDA; malondialdehyde, Cyt C; cytochrome C, Casp; caspase.

Overall, Figure 7 summarizes the mechanisms of the protection provided by the new saponin zygo-albuside **A** against MTX-induced testicular injury.

## 4. Materials and Methods

### 4.1. General Experimental Procedures

First, 1D and 2D NMR spectra (chemical shifts in ppm, coupling constants in Hz) were reported using Bruker Avance DRX 500 MHz spectrometers (Billerica, MA, USA). HRMS were measured through direct injection by Thermo Scientific UPLC RS Ultimate 3000-Q Exactive (Thermo Fisher Scientific, Waltham, MA, USA) hybrid quadrupole-Orbitrap mass spectrometer combined with high-performance quadrupole precursor selection with high resolution, accurate mass Orbitrap™ detection. Detection was performed in both negative and positive modes. Chromatographic separation was performed using columns packed with Sephadex LH-20 (0.25–0.1 mm, Pharmacia, Sigma-Aldrich, St. Louis, MI, USA) and silica gel 60 (0.04–0.063 mm). Precoated TLC plates (with silica gel 60 F254, 0.2 mm, Merck, Kenilworth, NJ, USA) and Whatman™ Grade 1 Chr Cellulose Chromatography Paper (Sigma-Aldrich, St. Louis, MI, USA) were used for detection of the chemical components. Spots were visualized using UV absorption at λ of 255 and 366 nm, in addition to spraying with *p*-anisaldehyde/H_2_SO_4_. Aniline phthalate reagent was used for visualization of sugar spots. Reference standards of *β* sitosterol (≥95%), *β* amyrin (phyproof^®^, ≥90%), ursolic acid (≥90), caffeic acid (≥98%), kaempferol (≥97%), quercetin (≥95%), rutin (phyproof^®^, ≥95%), D-glucose (≥99.5%), D-galactose (≥99%) were purchased from (Sigma-Aldrich, St. Louis, MI, USA).

### 4.2. Plant Material

*Z. album* was collected from Marsa Matrouh at the Northern Coast of the Mediterranean in Egypt during May 2019. It was identified at the Faculty of Science, Alexandria University. A voucher specimen was kept under registration number ZA-2019 in the herbarium of Pharmacognosy Department, Faculty of Pharmacy, Suez Canal University, Ismailia, Egypt.

### 4.3. Extraction and Isolation

First, 1.8 kg of the chopped small pieces of aerial parts of plant *Z. album* was extracted with methanol (3 × 2 L) at room temperature. The combined extract was concentrated under reduced pressure to give brownish green viscous crude extract (50 g).

The crude extract (45 g, suspended in 1 L. distilled water) was partitioned using *n*-hexane, CHCl_3_, EtOAc and *n*-BuOH to give five fractions (ZA-1–ZA-5) that were concentrated under reduced pressure. Fraction ZA-1 (100% *n*-hexane) (5 g) was applied on a silica gel column, 100% *n*-hexane was used for elution then gradient systems of *n*-hexane: EtOAc: MeOH, until reaching EtOAc: MeOH (50:50) to give ten subfractions (ZA-1-a to ZA-1-j). Subfraction (ZA-1-b) (610 mg) was chromatographed on silica gel and eluted by EtOAc: hexane starting with 5% EtOAc in hexane to 100% EtOAc followed by a Sephadex LH-20 column using CHCl_3_: MeOH (1:1) to finally acquire compound **1** (6.5 mg, white waxy substance). Subfraction (ZA-1-f) (550 mg) was similarly chromatographed on silica gel (the eluting system was EtOAc in hexane; 5% EtOAc in hexane to finally 100% EtOAc). Sephadex LH-20 column was applied for final purification using isocratic elution (CHCl_3_: MeOH; 1:1) to obtain compound **2** (3 mg, white powder).

Fraction ZA-2 (100% CHCl_3_) (6 g) was partitioned on a silica gel column and eluted with 10% EtOAc in *n*-hexane and then by gradient systems of *n*-hexane, EtOAc and MeOH until reaching 50% EtOAc in MeOH. This yielded nine subfractions (ZA-2-a to ZA-2-i). Subfraction (ZA-2-c) (990 mg) was applied on silica gel using gradient systems of EtOAc in hexane starting with 10% EtOAc in hexane to 100% EtOAc. Sephadex LH-20 column (with eluting mixture CHCl_3_: MeOH; 1:1) was set for final purification to acquire compound **3** (7 mg, creamy-white powder).

Fraction ZA-3 (100% EtOAc) (10 g) was added on a silica gel column, eluted with 100% CHCl_3_ and then by gradient systems (CHCl_3_ and MeOH until 50% MeOH in CHCl_3_); four subfractions (ZA-3-a to ZA-3-d) were obtained. Subfraction (ZA-3-a) (1.3 g) was purified on silica gel using elution by 100% CHCl_3_ then CHCl_3_: MeOH until 50% MeOH in CHCl_3_. This afforded three subfractions (ZA-3-a-1 to ZA-3-a-3). Subfraction (ZA-3-a-2) (250 mg) was treated similarly on silica gel (gradient systems: 100% CHCl_3_ then CHCl_3_: MeOH until 100% MeOH). Final purification was performed using Sephadex LH-20 column and CHCl_3_: MeOH (1:1) for isocratic elution to afford compound **4** (18 mg, colorless gum substance).

Subfraction (ZA-3-b) (1 g) was fractionated on silica gel column, eluted by gradient systems of 100% CHCl_3_ and MeOH until 50% MeOH in CHCl_3_. This afforded four subfractions (ZA-3-b-1 to ZA-3-b-4). Subfraction (ZA-3-b-1) (270 mg) was purified using silica gel and gradient systems of CHCl_3_: MeOH starting with 10% MeOH in CHCl_3_ until 100% MeOH. The purification was completed by a Sephadex LH-20 column and CHCl_3_: MeOH (1:1) for isocratic elution to obtain compound **5** (5 mg, yellowish powder).

Subfraction (ZA-3-c) (1 g) was treated in a similar was using silica gel column, eluted initially by 100% CHCl_3_, followed by gradient systems of CHCl_3_: MeOH until (1:1) CHCl_3_: MeOH to yield three subfractions (ZA-3-c-1 to ZA-3-c-3). Subfraction (ZA-3-c-3) (320 mg) was added on silica gel where gradient systems of CHCl_3_: MeOH were applied. (10% MeOH in CHCl_3_ until 100% MeOH). The last step in purification was performed using Sephadex LH-20 column and CHCl_3_:MeOH (1:1) to yield compound **6** (3 mg, yellowish powder).

Subfraction (ZA-3-d) (2.5 g) was chromatographed on silica gel using initially 100% CHCl_3_ and then with gradient systems of CHCl_3_:MeOH until (1:1) CHCl_3_:MeOH, which yielded four subfractions (ZA-3-d-1 to ZA-3-d-4). Subfraction (ZA-3-d-1) (350 mg) was added on silica gel using gradient systems (CHCl_3_:MeOH starting with 10% MeOH in CHCl_3_ until 100% MeOH), where two subfractions (ZA-3-d-1-a and ZA-3-d-1-b) were obtained. Subfraction (ZA-3-d-1-b) (180 mg) was purified on silica gel using gradient systems of CHCl_3_: MeOH starting with 10% MeOH in CHCl_3_ until 100% MeOH, and then, a Sephadex LH-20 column was applied for final purification using CHCl_3_:MeOH (1:1) to obtain compound **7** (65 mg, white powder).

Subfraction (ZA-3-d-4) (670 mg) was purified similarly using silica gel column and CHCl_3_ with gradual increase in MeOH (10% MeOH in CHCl_3_ until 100% MeOH); followed by a Sephadex LH-20 column using CHCl_3_: MeOH (1:1) to obtain pure compound **8** (60 mg, yellowish powder).

### 4.4. Acid Hydrolysis of Compound ***7***

Following the method described in [77], a quantity of 5 mg of compound **7** was hydrolyzed by 2 N HCl (1 mL) at 95 °C. The reaction mixture was then diluted with water and then extracted with CHCl_3_, and the recovered aglycone was subjected to PC and TLC co-chromatography with compound (**3**) and a standard sample of ursolic acid using benzene/toluene (1:4) [78], toluene/EtOAc/AcOH (6:3:1) [79] and 3% methanol in CHCl_3_ [80] as mobile phases.

The aqueous phase after usual work-up procedure yielded D-galactose identified by comparison with authentic sugars using silica gel TLC and propanol-H_2_O (8.5:1.5) as a developer [81], as well as PC applying *n*-butanol-benzene-pyridine-H2O (5:1:3:3) and PhOH satd. with H_2_O as mobile phases [82]. Sugar spots were visualized by aniline phthalate reagent.

### 4.5. Detection of the Sulfate Group

As described in [68], 5 mg of compound **7** was refluxed with 5 mL of HCl (2N) for 2 h and then neutralized with dil NaOH. The reaction mixture was then dried under vacuum. The residue was subjected to paper chromatography using 90% MeOH dried in air then sprayed with BaCl_2_ solution (100 mg in 50 mL of 70% MeOH), The paper was then air dried and finally sprayed with potassium rhodizoate solution (10 mg dissolved in 50 mL of 50% MeOH). A yellow color indicated the presence of sulfate group.

### 4.6. Spectroscopic Data of the Isolated Compounds

#### 4.6.1. Compound (**1**) (Rf = 0.25, 0.41 and 0.83; Mobile Phase: 25% EtOAc in Hexane, CHCl_3_/MeOH/AcOH (97:2:1) and Petroleum Ether/CHCl_3_/MeOH (49:50:1), Respectively [80,124,125]

^1^H NMR (C_5_D_5_N, 500 MHz): *δ*_H_ 1.01 (H-1a, m, H-1), 1.83 (H1b, m, H-1), 1.46 (2H, m, H-2), 3.52 (1H, dd, 5, 10, H-3), 2.21 (H-4a, m, H-4), 2.31 (H4b, m, H-4), 5.35 (1H, m, H-6), 1.85 (2H, m, H-7), 1.35 (1H, m, H-8), 0.85 (1H, m, H-9), 1.44 (H-11a, m, H-11), 1.49 (H11b, m, H-11), 1.13 (H-12a, m, H-12), 1.95 (H12b, m, H-12), 1.07 (1H, m, H-14), 1.18 (H-15a, m, H-15), 1.23 (H15b, m, H-15), 1.16 (2H, m, H-16), 1.00 (1H, m, H-17), 0.68 (3H, s, H-18), 0.92 (3H, s, H-19), 1.32 (1H, m, H-20), 0.84 (3H, d, *J* = 6.6, H-21), 1.28 (H-22a, m, H-22), 1.07 (H22b, m, H-22), 1.53 (2H, m, H-23), 1.53 (2H, m, H-23), 0.85 (1H, m, H-24), 1.64 (1H, m, H-25), 0.80 (3H, d, *J* = 6.6, H-26), 0.84 (3H, d, *J* = 7.2,H-27), 1.18 (H-28a, m, H-28), 1.23 (H28b, m, H-28), 0.80 (3H, t, *J* = 7.2, H-29).

^13^C NMR (MeOD, 125 MHz): *δ_C_* 11.9 (C-29), 12.0 (C-24), 18.8 (C-28), 19.4 (C-19), 19.9 (C-27), 21.1 (C-11, C-26), 23.1 (C-23), 24.3 (C-15), 26.0 (C-21), 28.3 (C-16), 29.1 (C-25), 31.9 (C-2, C-7, C-8), 33.9 (C-20), 36.2 (C-18), 36.5 (C-10), 37.3 (C-1, C-12), 42.2 (C-13), 42.5 (C-4), 45.8 (C-22), 50.1 (C-9), 56.0 (C-17), 56.8 (C-14), 71.8 (C-3), 121.7 (C-6), 140.7 (C-5).

#### 4.6.2. Compound (**2**) (R_f_ = 0.39, 0.57 and 0.58; Mobile Phase: 25% EtOAc in Hexane, CHCl_3_/MeOH (97:3) and Toluene/MeOH (9:1) and, Respectively [80,126,127]

^1^H NMR (CDCl_3_, 300 MHZ) at *δ*; 0.69, 0.76, 0.85, 0.87, 0.90, 0.95, 1.16, 1.19 (each 3H, s, C-25, C-23, C-30, C-29, C-24, C-26, C-27, C-28), 3.62 (dd, *J =* 10, 5 Hz, H-3) 5.73 (d, *J =* 7.2 Hz, H-12).

^13^C NMR (CDCl_3_, 125 MHz): *δ*_C_ 14.4 (C-24), 14.4 (C-25), 17.3 (C-26), 17.4 (C-6), 20.0 (C-11), 22.7 (C-30), 22.7 (C-27), 24.5 (C-16), 25.6 (C-15), 26.3 (C-2), 27.0 (C-23), 28.6 (C-28), 29.9 (C-20), 31.8 (C-7), 33.3 (C-17), 33.2 (C-29), 34.4 (C-21), 35.3 (C-10), 35.2 (C-22), 37.1 (C-4), 37.5 (C-1), 39.9 (C-8), 41.8 (C-14), 47.3 (C-9), 48.5 (C-19), 49.5 (C-18), 54.5 (C-5), 78.2 (C-3), 122.9 (C-12), 145.9 (C-13).

#### 4.6.3. Compound (**3**) (R_f_ = 0.30, 0.64 and 0.71; Mobile Phase: Benzene/Toluene (1:4), Toluene/EtOAc/AcOH (6:3:1) and 3% MeOH in CHCl_3_, Respectively [78,79,80]

^1^H NMR (C_5_D_5_N, 500 MHz): *δ*_H_ 1.03 (H-1a, m, H-1), 1.57 (H1b, m, H-1), 1.81 (2H, m, H-2), 3.44 (1H, dd, 5, 10, H-3), 0.87 (1H, m, H-5), 1.36 (H-1a, m, H-6), 1.57 (H1b, m, H-6), 1.36 (H-1a, m, H-7), 1.57 (H1b, m, H-7), 1.67 (1H, m, H-9), 1.96 (2H, m, H-11), 5.48 (1H, s, H-12), 1.27 (H-1a, m, H-15), 2.13 (H1b, t, 10, H-15), 2.14 (1H, t, 10, H-16), 2.02 (1Hb, m, H-16), 2.63 (1H, d, 10, H-18), 1.47 (1H, m, H-19), 1.05 (1H, m, H-20), 1.44 (1Ha, m, H-21), 1.52 (1Hb, m, H21), 1.94 (2H, m, H-22), 1.28 (3H, s, H-23), 1.03 (3H, s, H-24), 0.94 (3H, s, H-25), 1.05 (3H, s, H-26), 1.27 (3H, s, H-27), 1.03 (3H, s, H-29), 0.97 (3H, d, 10, H-30).

^13^C NMR (C5D5N, 125 MHz): *δ*_C_ 15.5 (C25), 16.4 (C24), 17.2 (C26, C29), 18.6 (C-6), 21.2 (C30), 23.6 (C27), 23.7 (C-11), 28.1 (C-2), 28.5 (C23), 28.6 (C-15), 32.9 (C21), 33.1 (C-7), 37.0 (C22), 37.1 (C-10), 38.7 (C20), 39.2 (C-1, C19), 39.5 (C-4), 41.8 (C-8), 42.3 (C-14), 47.1 (C-16), 47.8 (C17), 47.9 (C-9), 53.3 (C18), 55.6 (C-5), 77.9 (C-3), 122.3 (C-12), 144.6 (C-13), 179.9 (C28).

#### 4.6.4. Compound (**4**) (R_f_ = 0.35, 0.79 and 0.96; Mobile Phase: Toluene/EtOAc/HCOOH (6:3:1), *n*-Butanol/AcOH/H_2_O (4:1:5) and EtOAc/AcOH/HCOOH/H_2_O (100:11:11:26), Respectively [79,82,128]

^1^H NMR (MeOD, 500 MHz): *δ*_H_ 7.45 (1H, d, *J* = 16.0 Hz, H-7), 6.94 (1H, d, *J* = 1.5 Hz, H-2), 6.85 (1H, dd, *J* = 8.0, 1.5 Hz, H-6), 6.69 (1H, d, *J* = 8.0 Hz, H-5), 6.14 (1H, d, *J* = 16.0 Hz, H-8).

^13^C NMR (MeOD, 125 MHz): *δ*_C_ 126.4 (C-1), 114.2 (C-2), 145.6 (C-3), 148.1 (C-4), 115.1 (C-5), 121.5 (C-6), 145.4 (C-7), 113.7 (C-8), 169.7 (C-9).

#### 4.6.5. Compound (**5**) (R_f_ = 0.26, 0.55 and 0.58; Mobile Phase: Toluene/EtOAc/HCOOH (7:3:0.1), Forestal = conc. HCl/AcOH/H_2_O (3:30:10) and PhOH/H_2_O (3:1), Respectively [82,129]

^1^H NMR (DMSO-d6, 500 MHz): *δ*_H_ 6.18 (1H, d, *J* = 2 Hz, H-6), 6.44 (1H, s), 8.04 (1H, dd, *J* = 2.8, 8.5 Hz, H-2′), 6.94 (1H, dd, *J* = 2.7, 10 Hz, H-5′), 8.04 (1H, dd, *J* = 2.7, 11.6 Hz, H-6′), 6.94 (1H, dd,2.7, 8.5, H-3′).

^13^C NMR (DMSO-d_6_, 125 MHz): *δ*_C_ 147.2 (C-2), 136.1 (C-3), 176.3 (C-4), 161.2 (C-5), 98.7 (C-6), 164.4 (C-7), 93.9 (C-8), 156.6 (C-9), 103.5 (C-10), 122.1 (C-1′), 129.9 (C-2′), 115.9 (C-3′), 159.6 (C-4′), 115.9 (C-5′), 129.9 (C-6′).

#### 4.6.6. Compound (**6**) (R_f_ = 0.29, 0.41 and 0.9; Mobile Phase: PhOH/H_2_O (3:1), Forestal = conc. HCl/AcOH/H_2_O (3:30:10) and EtOAc/AcOH/H_2_O (7.5:1.5:1.5), Respectively [80,130]

^1^H NMR (DMSO-d_6_, 500 MHz): *δ*_H_ 6.20 (1H, d, *J* = 2 Hz, H-6), 6.42 (1H, d, *J* = 2 Hz, H-8), 7.70 (1H, d, *J* = 2.2 Hz, H-2′), 6.90 (1H, d, *J* = 8.5 Hz, H-5′), 7.56 (1H, dd, *J* = 8.5, 2.2 Hz, H-6′), 9.41 (1H, s, H-4′), 9.35 (1H, s, H-3′), 12.49 (1H, s, H-3), 12.52 (1H, s, H-5), 12.49 (1H, s, H-7).

^13^C NMR (DMSO-d_6_, 125 MHz): *δ*_C_ 147.2 (C-2), 136.2 (C-3), 176.2 (C-4), 156.6 (C-5), 98.7 (C-6), 164.3 (C-7), 93.8 (C-8), 161.6 (C-9), 103.5 (C-10), 122.5 (C-1′), 116.0 (C-2′), 145.5 (C-3′), 148.1 (C-4′), 115.5 (C-5′), 120.8 (C-6′).

#### 4.6.7. Compound (**7**) (R_f_ for the aglycone (R_f_ = 0.30, 0.64 and 0.71 Mobile Phase: Benzene/Toluene (1:4), Toluene/EtOAc/AcOH (6:3:1) and 3% MeOH in CHCl_3_, Respectively. [78,79,80]. R_f_ for the Sugar Part = 0.21 0.38 and 0.39; Mobile Phase: *n*-Butanol/Benzene/Pyridine/H_2_O (5:1:3:3), PhOH satd. with H_2_O and Propanol/H_2_O (8.5:1.5), respectively) [79,80]

White powder; HRMS: *m/z* 697.2885 [M-H]^−^; ^1^H NMR (MeOH, 500 MHz) and ^13^C NMR (MeOH, 125 MHz) spectral data, see Table 1.

#### 4.6.8. Compound (**8**) (R_f_ = 0.24, 0.28 and 0.45; Mobile Phase: EtOAc/AcOH/HCOOH/H_2_O (100:10:10:18), EtOAc/AcOH/H_2_O (7.5:1.5:1.5) and EtOAc/AcOH/HCOOH/H_2_O (100:11:11:26) [130,131,132]

^1^H NMR (DMSO-d_6_, 500 MHz): *δ*_H_ 6.20 (1H, d, *J* = 2 Hz, C6-H), 6.40 (1H, d, *J* = 2 Hz, C8-H), 7.55 (1H, s, C2′-H), 6.87 (1H, d, *J* = 8.5 Hz, C5′-H), 7.55 (1H, s, C6′-H), 12.59 (1H, s, C5-OH), 5.35 (1H, s, H1″), 3.40 (1H, m, H2″, H5″), 3.30* (1H, m, H3″), 3.28* (1H, m, H4″), 3.41* (1H, m, H6″), 3.84 (1H, m, H6″), 5.15 (1H, d, *J* = 1.9, H1‴), 3.62* (1H, m, 2‴), 3.60* (1H, m, 3‴), 3.30* (1H, m, 4‴), 3.42* (1H, m, 5‴), 1.00 (3H, d, *J* = 6.1, CH_3_-6‴).

^13^C NMR (DMSO-d_6_, 125 MHz): 18.2 (C6‴), 67.4 (C6″), 68.7 (C5‴), 70.4 (C4‴), 70.8 (C2‴), 71.0 (C3‴), 72.3 (C4″), 74.5 (C2″), 76.3 (C5″), 76.8 (C3″), 94.1 (C-8), 99.2 (C-6), 101.2 (C1‴), 101.6 (C1″), 104.4 (C-10), 115.7 (C-2′), 116.7 (C-5′), 121.6 (C-6′), 122.1 (C-1′), 133.7 (C-3), 145.2 (C-3′), 148.8 (C-4′), 157.1 (C-2, C-5), 161.6 (C-9), 164.5 (C-7), 177.9 (C-4).

### 4.7. In Vitro Antioxidant Assay of Compound ***7***

#### 4.7.1. DPPH Radical Scavenging Activity

The method was applied as previously published [133]. The measurements proceeded as the average of triplicates. The percentage of inhibition (PI) of the DPPH radical was deemed based on the formula:

PI = [{(*A*C − *A*T)/*A*C} × 100] (1)

where *A*C = absorbance of the control at t = 0 min, and *A*T = absorbance of the compound **7** + DPPH at t = 16 min.

IC_50_ is the concentration needed to 50%; DPPH radical scavenging activity was calculated from graphic plots of the dose response curve using Graphpad Prism software (San Diego, CA, USA) [134].

#### 4.7.2. Hydrogen Peroxide Radical (H_2_O_2_) Scavenging Activity

The test was performed based on the previously published procedure [135]. BHT was the reference standard. The H_2_O_2_ radical scavenging percentage of compound **7** was estimated by the following equation:

H_2_O_2_ radical scavenging percentage = [(A_blank_ − A_sample_)/A_blank_] × 100 g extract

IC_50_ was estimated from graphic plots of the dose–response curve using Graphpad Prism software (San Diego, CA, USA).

#### 4.7.3. Total Antioxidant Capacity (TAC)

Total antioxidant capacity of compound **7** was evaluated by the phosphomolybdate complex method [136]. TAC was measured (as mg equivalents gallic per 100 g compound **7**) using the standard gallic graph.

### 4.8. In Vivo Investigation of the Protective Effects of Z. album Extract, Compound ***7*** and Compound ***8*** against MTX-Induced Testicular Injury

#### 4.8.1. Preliminary Assessment of the Liver and Kidney Toxicity

A total of 20 mice (20–30 g) were randomly divided into 4 groups (5 mice each). The first group received the vehicle (distilled water:DMSO (2:1)) and was considered the normal group. The other three groups received Z. album extract (100 mg/kg), zygo-albuside **A** (10 mg/kg) and rutin (10 mg/kg), respectively. The determined doses were administered orally for 7 consecutive days. Blood samples were collected from the mice after 7 days and serum was separated. Serum levels of the liver function enzymes ALT and AST were assessed by colorimetric kits; AL1031 and AS1061, respectively (Biodiagnostic, Giza, Egypt). Similarly, the serum levels of the kidney markers urea and creatinine were also determined calorimetrically; UR2110 and CR1250, respectively (Biodiagnostic, Giza, Egypt).

#### 4.8.2. Animals and Treatment (the Principle In Vivo Study)

The current in vivo investigative study was performed on 40 male Swiss albino mice of the same age with an initial average body weight of approximately 20–30 g. The minimum number of animals required to achieve statistical significance was used, and every effort was made to minimize animal suffering. These mice were bred at the “Animal Sector” of the Egyptian Biological Products and Vaccines Organization (Vacsera, Giza, Egypt). The mice were kept in aluminum cages at controlled room temperature (25 °C). All animal handling was approved by the Ethical Committee of the Faculty of Pharmacy, Suez Canal University (202010PHDA_1_). After a two-week acclimation period, the animals were randomly divided into five groups (8 mice each). The mice in the first group received a mixture of distilled water and DMSO (2:1) as vehicle orally every day for ten days and served as the NC group (Group I). In the remaining mice, testicular damage was induced on the fifth day by a single intraperitoneal (i.p.) dose of 20 mg/kg methotrexate (MTX) [137]. These mice were randomly divided into four groups: Group II (MTX positive control group): Mice received only MTX; Group III (crude extract + MTX): Mice were received 100 mg/kg crude extract of *Z. album* [138]; Group IV (Zygo-albuside **A** + MTX): Mice received 10 mg/kg zygo-albuside **A** [139]; Group V (Rutin + MTX): Mice received 10 mg/kg rutin [140].

*Z. album* crude extract, zygo-albuside **A**, and rutin were administered daily by oral gavage for the total period of the experiment (i.e., 10 days).

#### 4.8.3. Tissue Collection

Mice were sacrificed by decapitation under ketamine anesthesia at the end of the 10-day treatment. Blood samples were collected for serum separation in plain vacutainers. Testis were removed, weighed, and decapsulated, and a portion of the tissue was stored at −80 °C for biochemical analysis. Another part of the testis was placed in formalin solution (10%) for histopathological studies.

#### 4.8.4. Biochemical Analysis

##### Measurement of Testosterone

The level of testosterone levels (cat. no. MBS263193) was determined in serum by ELISA kit (MyBioSource, San Diego, CA, USA).

##### Determination of the Level of Oxidative Stress in the Testis

One gram of testis was homogenized in 2 mL of ice-cold phosphate-buffered saline (PBS) lyses buffer (pH = 7.4) using a disintegrator (Ultra-Turrax homogenizer, Darmstadt, Germany). The homogenate was then centrifuged at 3000× *g* for 15 min, and the supernatant obtained was frozen in aliquots at −80 °C until analysis. The levels of MDA, SOD, and CAT in the testis were determined using mice-specific ELISA kits (San Diego, CA, USA; Cat. No. MBS741034, MBS034842, and MBS160589, respectively).

##### Determination of Testicular Inflammation and Apoptosis Mediators

Levels of IL-1β, IL-6, Bax, and Bcl-2 were determined in the homogenate samples of testicular tissue by mice-specific ELISA kits according to the manufacturer’s instructions ELISA kit (MyBioSource, San Diego, CA, USA; cat. no. MBS701092, MBS762321, MBS763832-, and MBS2512543, respectively).

##### Determination of Testicular Expression of NF-κB, TNF-α, p53, and miR-29a by Quantitative Real-Time Polymerase Chain Reaction

The Qiagen miRNeasy Mini Kit (cat. no. 217004) was used to separate total RNA, including miRNA, from testicular tissue (Qiagen, Hilden, Germany). NanoDrop spectrophotometer was used to determine the concentration and purity of the extracted RNA (Thermo Fisher Scientific, Waltham, MA, USA).

Expression levels of NF-κB, TNF-α, p53, and miR-29a were measured in testicular tissue using GoTaq^®^ 1-Step RT-qPCR technology (Promega, Madison, WI, USA). The endogenous control for NF-κB, TNF-α, and p53 was β-Actin, whereas miR-29a was controlled by small nuclear RNA U6B (RNU6B). The primers and annealing temperatures are listed in (Table S2). For the experiment, 4 µL of RNA template, 0.4 µL of GoScriptTM RT mix for 1-step RT-qPCR, 1 µL of forward and reverse primers, 10 µL of GoTaq^®^ qPCR master mix, 0.31 µL of additional CXR reference dye, and 3.29 µL of nuclease-free water were used. Cycles included 15 min of reverse transcription at 37 °C, 10 min of reverse transcriptase enzyme inactivation at 95 °C, and 40 cycles of denaturation at 95 °C for 10 s, annealing for 30 s, and extension at 72 °C for 30 s. The StepOnePlusTMReal-Time PCR Thermal Cycler was used for all real-time PCR experiments (Applied Biosystems, Waltham, MA, USA). The ΔΔCt and fold change values were determined, and the results were expressed as the mean fold change compared with the NC group.

#### 4.8.5. Histological Analysis

For histological examination, testicular tissue was cut into tiny slices and fixed in 10% buffered formalin before being treated with a graded ethanol series, embedded in kerosene, and finally cut into 2–3 m thick slices. Hematoxylin and eosin (H&E) stains were used for regular histological assessment. An Olympus microscope (Shinjuku, Japan) equipped with a spot digital camera and MATLAB software computer program was used for observation, and specimens were photographed. To evaluate the histopathological changes, the Johnsen scoring system was used to calculate the degree of seminiferous tubules. Depending on the presence of spermatogenic cells, the system was scored as the followings: 1, absence of seminiferous epithelium; 2, no germinal cells; 3, only spermatogonia; 4, there are no spermatozoa or spermatids and only a few spermatocytes; 5, no spermatozoa or spermatids, but many spermatocytes; 6, there are no spermatozoa, no late spermatids, and only a few early spermatids; 7, no spermatozoa, no late spermatids, but plenty of early spermatids; 8, spermatozoa per tubule, with few late spermatids; 9, somewhat disrupted spermatogenesis, several late spermatids, and disordered epithelium; 10, full spermatogenesis [98].

#### 4.8.6. Statistical Analysis

Data were analyzed using Statistical Package for Social Sciences (SPSS) software, version 17 (Chicago, IL, USA) SPSS. Results were presented as mean ± SD. Significant differences between treatment effects were determined by one-way analysis of variance (ANOVA) followed by Bonferroni’s post hoc multiple comparison test, with statistical significance level set at <0.01.

## 5. Conclusions

The current study afforded the isolation and structure elucidation of a new saponin; zygo-albuside **A**, along with seven known compounds from *Z. album* areal parts. The study demonstrated for the first time that administration of isolated *Z. album* crude extract and its isolated compounds, zygo-albuside **A** and rutin, significantly attenuated testicular damage through suppressing apoptosis via inhibition of the p53 pathway and downregulation of miRNA-29a. The investigated *Z. album* extract and its isolated compounds zygo-albuside **A** and rutin also reduced inflammation via downregulation of NF-κB and reduction of proinflammatory cytokines and enhanced the antioxidant capacity by increasing SOD and CAT. The current study provides a new promising natural product, zygo-albuside **A**, as a potential counteracting agent against MTX side effects on testis. Complete assessment of the toxicity profile of the newly isolated compound zygo-albuside **A** is required in future studies to indicate its therapeutic index.

## Figures and Tables

**Table 1:** ^1^H (500 MHz) and ^13^C (125 MHz) NMR spectroscopic data of compound **7** (MeOH, *δ* in ppm, *J* in Hz).

No.	*δ_C_*	*δ_H_* (Int., Mult., *J*_Hz_)	No.	*δ_C_*	*δ_H_* (Int., Mult., *J*_Hz_)
1	39.0	1.10 (1H, m) 1.62 (1H, m)	19	38.6	1.11 (1H, m)
2	27.5	1.25 (1H, m) 1.42 (1H, m)	20	38.5	0.95 (1H, m)
3	74.9	3.64 (1H, dd, 5, 10)	21	30.8	1.02 (1H, m), 1.62 (1H, m)
4	38.9	-	22	37.7	1.41 (1H, m), 2.06 (1H, m)
5	55.6	0.95 (1H, m)	23	28.4	1.03 (3H, S)
6	19.9	0.95 (1H, m) 1.35 (1H, m)	24	19.9	0.93 (3H, S)
7	38.6	1.62 (1H, m) 1.73 (1H, m)	25	17.5	0.90 (3H, S)
8	40.4	-	26	19.9	1.00 (3H, S)
9	47.1	1.19 (1H, m)	27	13.6	1.10 (3H, S)
10	39.0	-	28	172.6	-
11	25.8	1.96 (1H, m), 2.15 (1H, m)	29	17.6	1.11 (1H, d, 10)
12	126.1	5.35 (1H, m)	30	21.4	0.94 (1H, d, 8)
13	138.5	-	1′	86.8	4.75 (1H, d, 10)
14	57.1	-	2′	78.5	4.07 (1H, dd, 5, 10)
15	28.4	1.03 (1H, m) 1.11 (1H, m)	3′	73.3	3.89 (1H, t, 5)
16	25.8	1.39 (1H, m) 2.16 (1H, m)	4′	68.9	4.08 (1H, m)
17	47.2	-	5′	74.6	3.65 (1H, m)
18	54.8	1.79 (1H, m)	6′	60.3	3.65 (1H, m) 3.89 (1H, m)

**Table 2:** In vitro antioxidant activities of compound **7** by DPPH, H_2_O_2_ and TAC assays.

Sample	DPPH (IC_50_ in µg/mL)	H_2_O_2_ (IC_50_ in µg/mL)	TAC (mg GAE/g)
Compound **7**	45.41 ± 2.65	65.16 ± 3.22	29.83 ± 2.19
Ascorbic acid	10.64 ± 0.82	NT	71.28 ± 4.34

NT, not tested. Data are expressed as mean ± SD of three independent values.

## Data Availability

Not applicable.

## Supplementary Materials

The supporting information can be downloaded at: https://www.mdpi.com/article/10.3390/ijms231810799/s1.
