# Supplementary material for: Zygo-Albuside A: New Saponin from Zygophyllum album L. with Significant Antioxidant, Anti-Inflammatory and Antiapoptotic Effects against Methotrexate-Induced Testicular Damage
Source: Int J Mol Sci. 2022 Sep 16;23(18):10799. doi: 10.3390/ijms231810799 (PMC9501557; doi:10.3390/ijms231810799)
Supplement: Supplementary file 1 [file ijms-23-10799-s001.zip › ijms-1891169-supplementary.pdf]

Zygo-Albuside A: New Saponin from *Zygophyllum album* L. with Significant Antioxidant, Anti-Inflammatory and Antiapoptotic Effects against Methotrexate-Induced Testicular Damage

Table of Contents

Figure S1: ^{1}H-NMR spectrum of Compound 1 (500 MHz, $CDCL_3$). 2
Figure S2: ^{13}C-NMR spectrum of Compound 1 (125 MHz, $CDCL_3$). 3
Figure S3: ^{1}H-NMR spectrum of Compound 2 (500 MHz, $CDCL_3$). 4
Figure 4: ^{13}C-NMR spectrum of Compound 2 (125 MHz, $CDCL_3$). 5
Figure S5: ^{1}H-NMR spectrum of Compound 3 (500 MHz, C_5D_5N). 6
Figure S6: ^{13}C-NMR spectrum of Compound 3 (125 MHz, C_5D_5N). 7
Figure S7: ^{1}H-NMR spectrum of Compound 4 (500 MHz, MeOH). 8
Figure S8: ^{13}C-NMR spectrum of Compound 4 (125 MHz, MeOH). 9
Figure S9: ^{1}H-NMR spectrum of Compound 5 (500 MHz, DMSO-d6). 10
Figure S10: ^{13}C-NMR spectrum of Compound 5 (125 MHz, DMSO-d6). 10
Figure S11: ^{1}H-NMR spectrum of Compound 6 (500 MHz, DMSO-d6). 11
Figure S12: ^{13}C-NMR spectrum of Compound 6 (125 MHz, DMSO-d6). 11
Figure S13: HRMS of Compound 7 12
Figure S14: ^{1}H-NMR spectrum of Compound 7 (500 MHz, MeOH). 13
Figure S15: Partial expansion of ^{1}H-NMR spectrum of Compound 7 (500 MHz, MeOH). 14
Figure S16: Partial expansion of ^{1}H-NMR spectrum of Compound 7 (500 MHz, MeOH). 15
Figure S17: ^{13}C-NMR spectrum of Compound 7 (125 MHz, MeOH). 16
Figure S18: Partial expansion of ^{13}C-NMR spectrum of Compound 7 (125 MHz, MeOH). 17
Figure S19: Partial expansion of ^{13}C-NMR spectrum of Compound 7 (125 MHz, MeOH). 18
Figure S20: Partial expansion of ^{13}C-NMR spectrum of Compound 7 (125 MHz, MeOH). 19
Figure S21: Partial expansion of ^{13}C-NMR spectrum of Compound 7 (125 MHz, MeOH). 20
Figure S22: DEPT 135 of Compound 7 (125 MHz, MeOH). 21
Figure S23: COSY of Compound 7 22
Figure S24: Partial expansion of COSY of Compound 7. 22
Figure S25: HMBC of compound 7 23
Figure S26: Expansion of HMBC of compound 7. 24
Figure S27: HSQC of compound 7 25
Figure S28: Expansion of HSQC of compound 7. 26
Figure S29: Expansion of HSQC of compound 7. 27
Figure S30: ^{1}H-NMR spectrum of Compound 8 (500 MHz, DMSO-d6). 28
Figure 31: ^{13}C-NMR spectrum of Compound 8 (125 MHz, DMSO-d6). 29
Table S1: Liver enzymes and kidney markers in the groups of the preliminary study……………......29

Table S2: Primer sequences and annealing temperatures for genes amplification…………………30

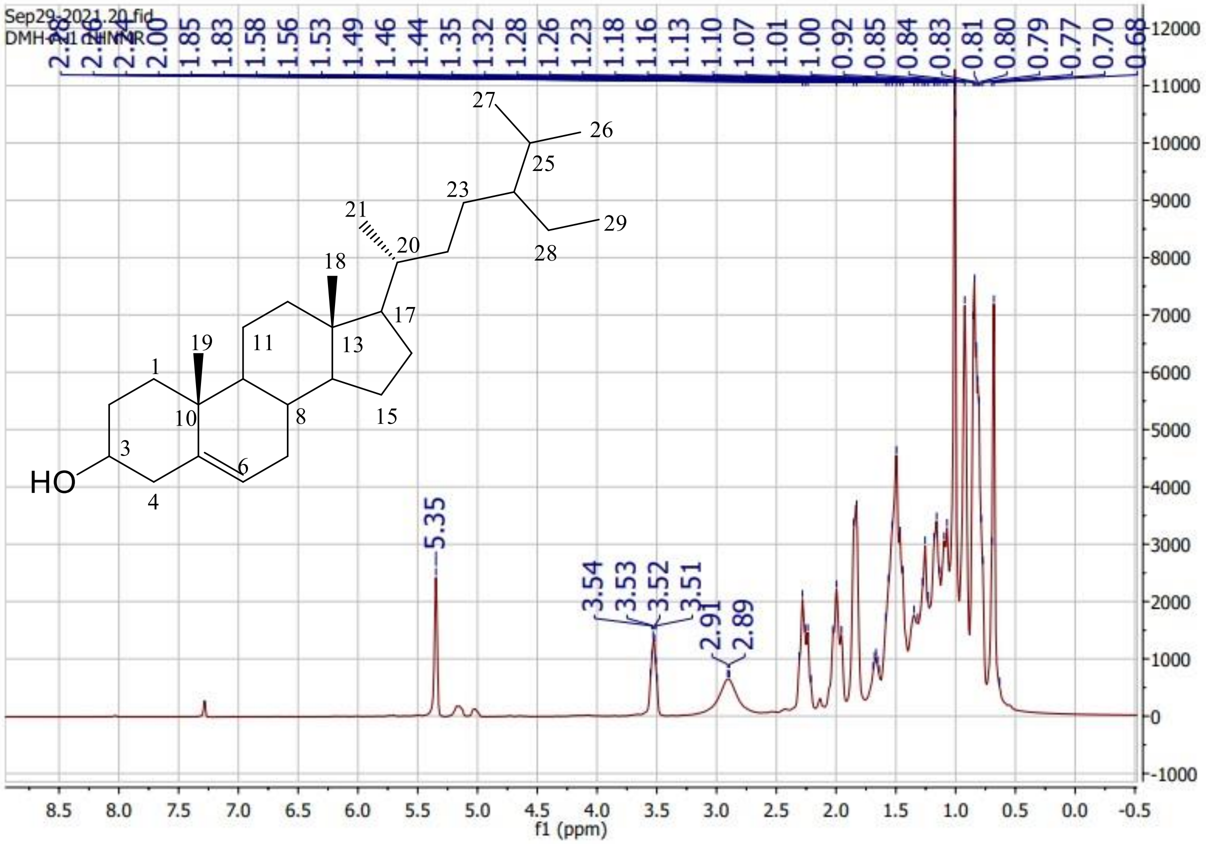

Figure S1: ¹H-NMR spectrum of Compound 1 (500 MHz, CDCL$_3$).

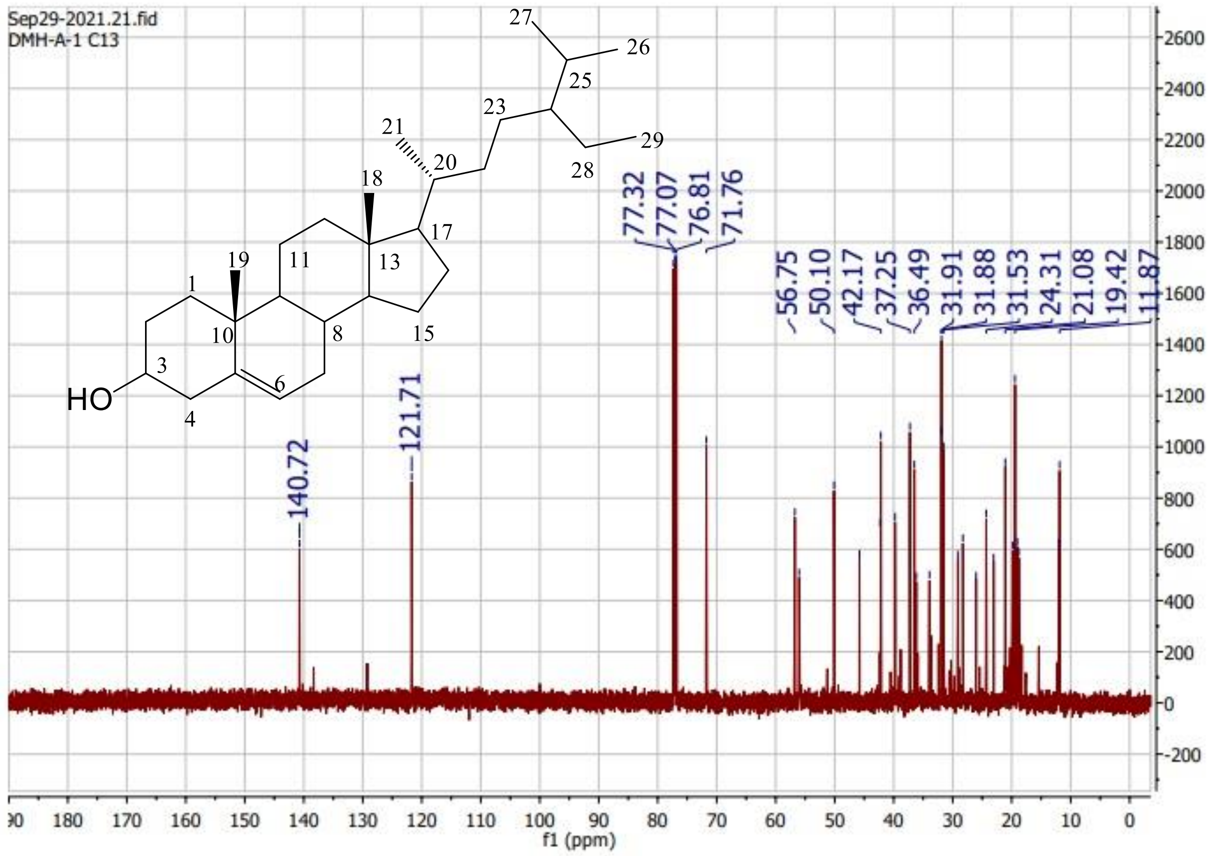

Figure S2: ^{13}C-NMR spectrum of Compound 1 (125 MHz, CDCL$_3$).

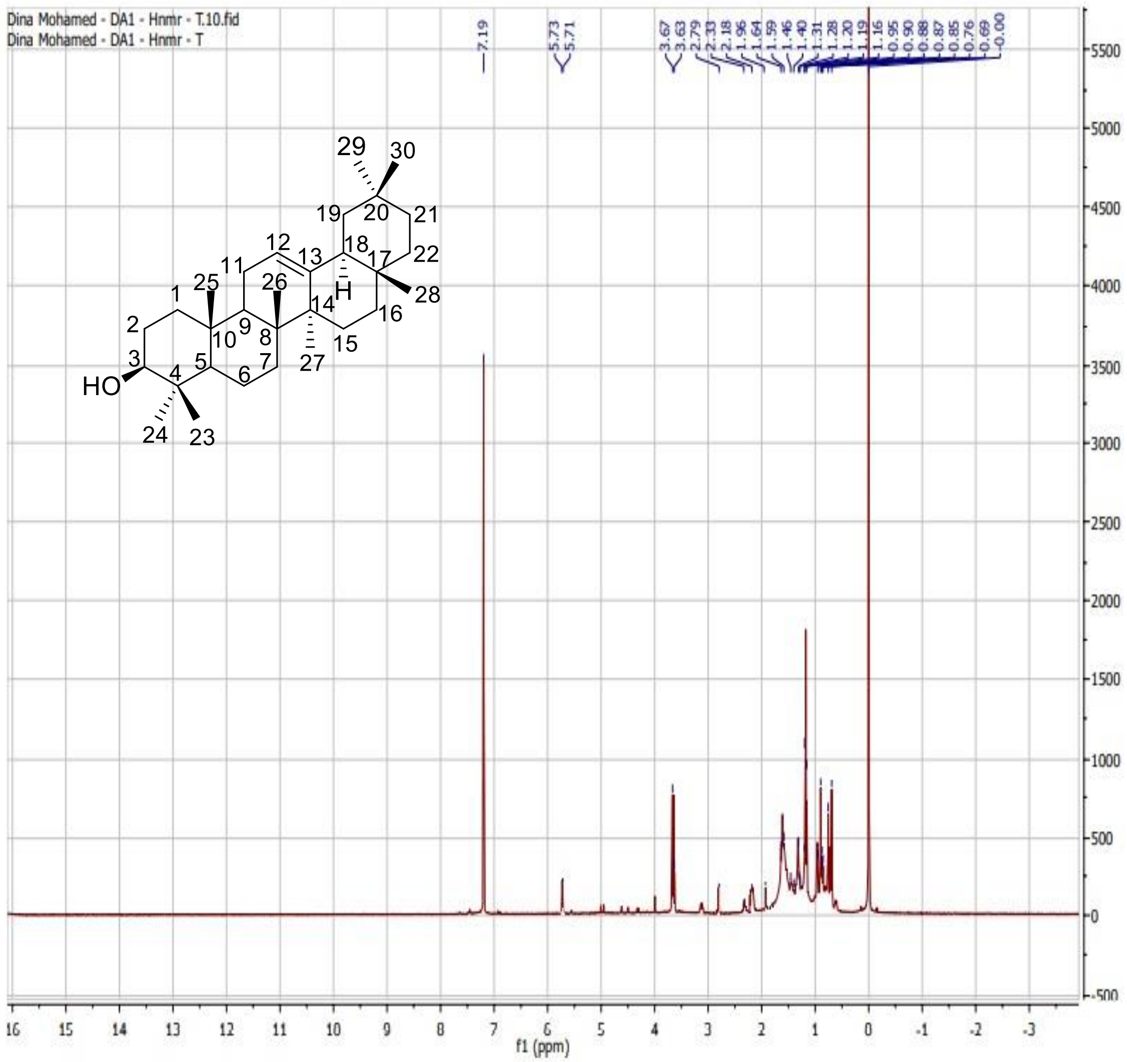

Figure S3: ^{1}H-NMR spectrum of Compound 2 (500 MHz, CDCL$_3$).

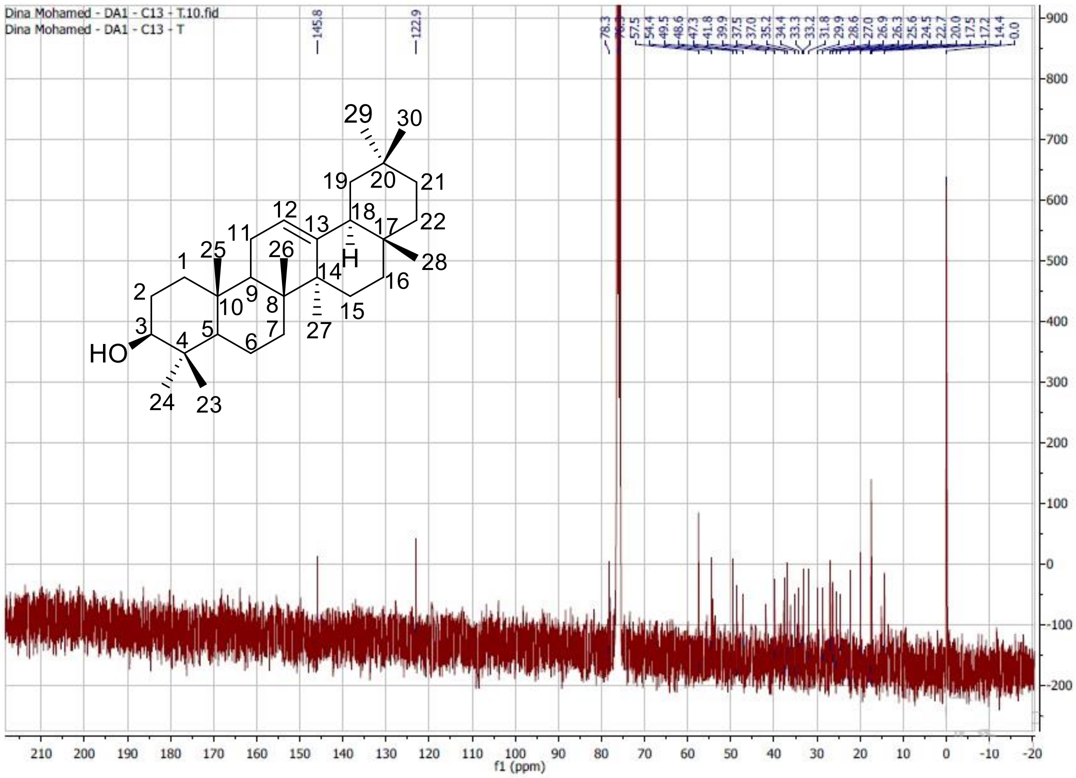

Figure 4: ^{13}C-NMR spectrum of Compound 2 (125 MHz, CDCL$_3$).

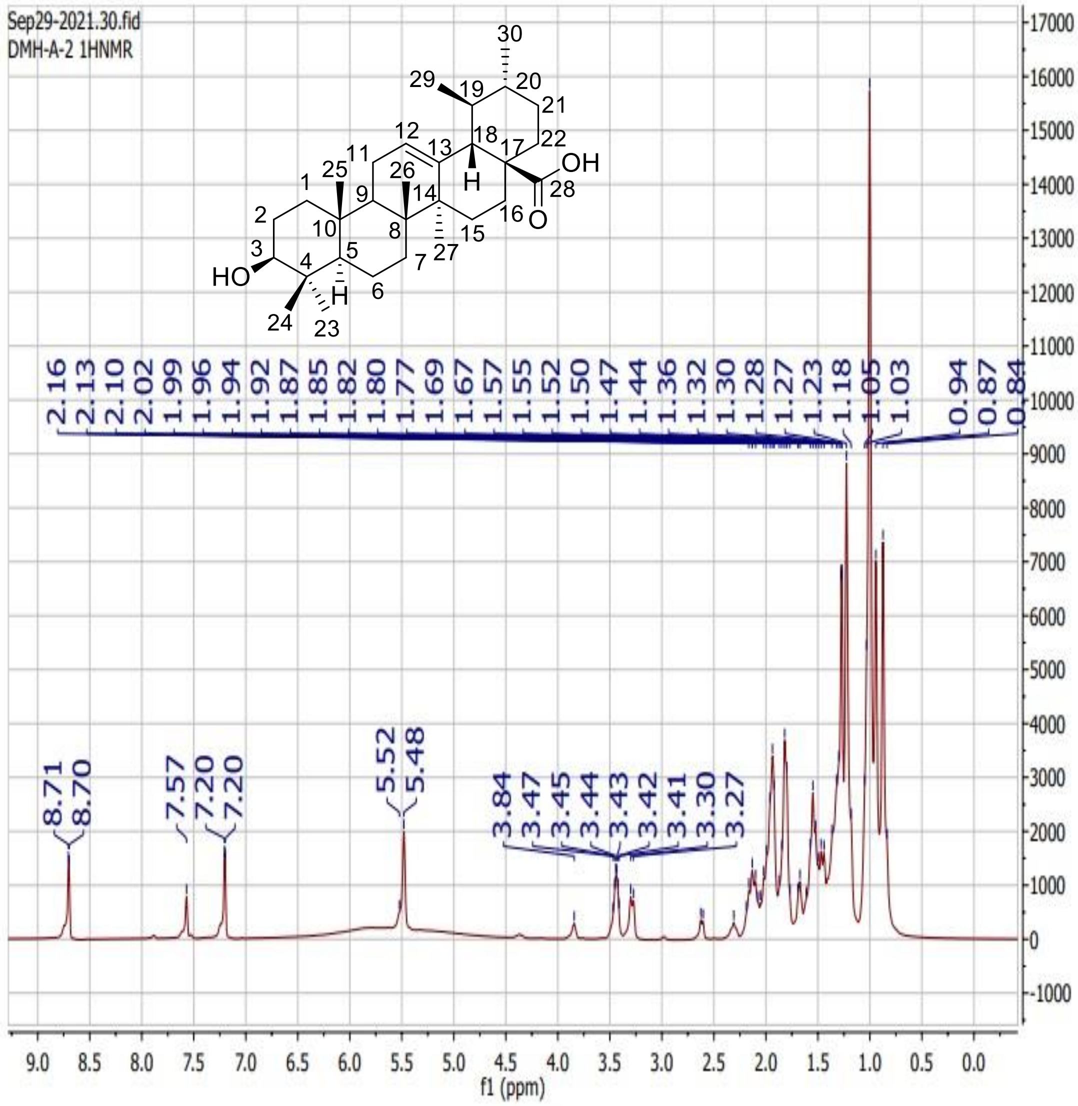

Figure S5: ^{1}H-NMR spectrum of Compound 3 (500 MHz, C_5D_5N).

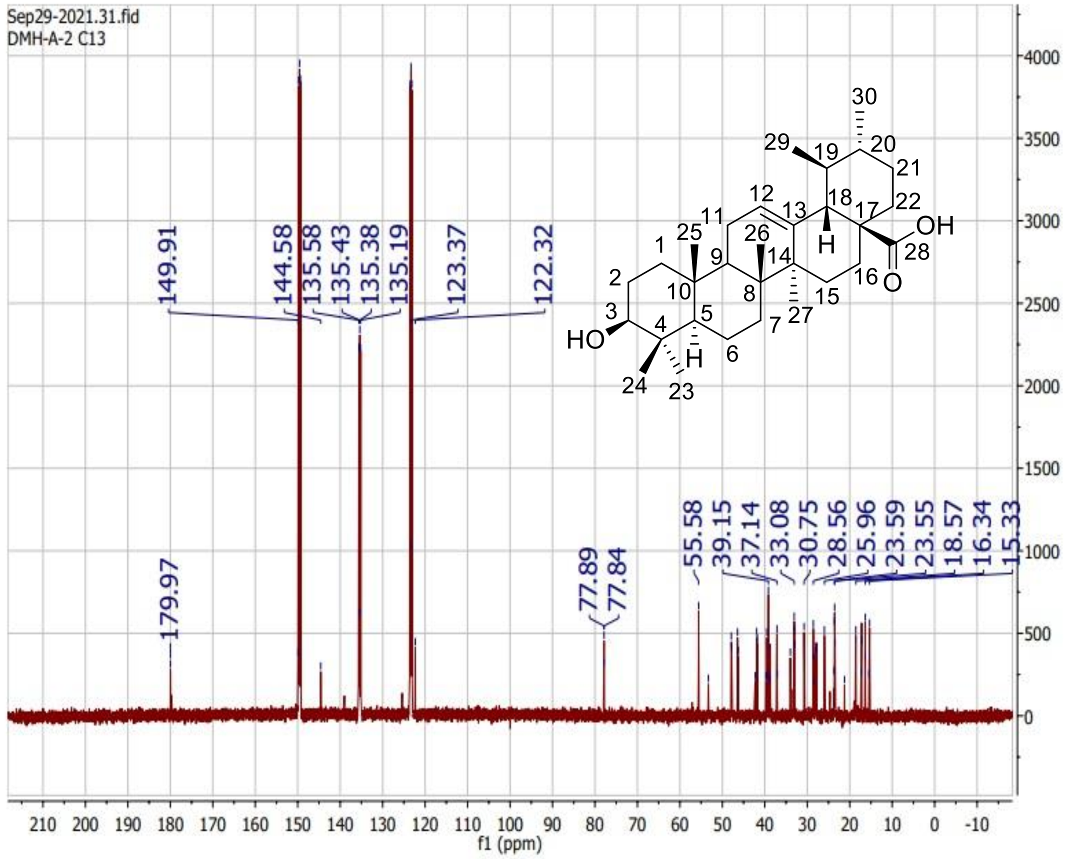

Figure S6: ^{13}C-NMR spectrum of Compound 3 (125 MHz, C_5D_5N).

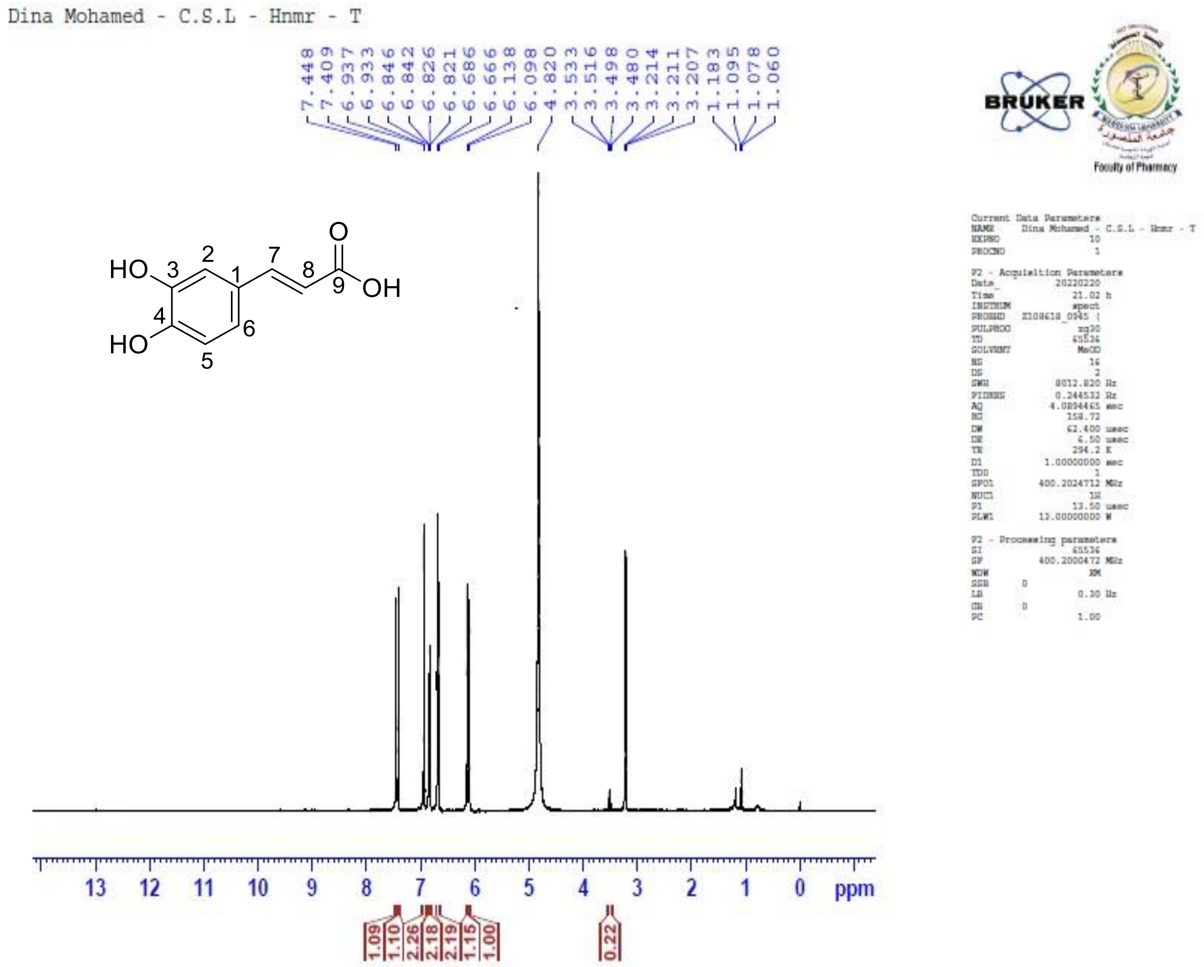

Figure S7: ^{1}H-NMR spectrum of Compound 4 (500 MHz, MeOH).

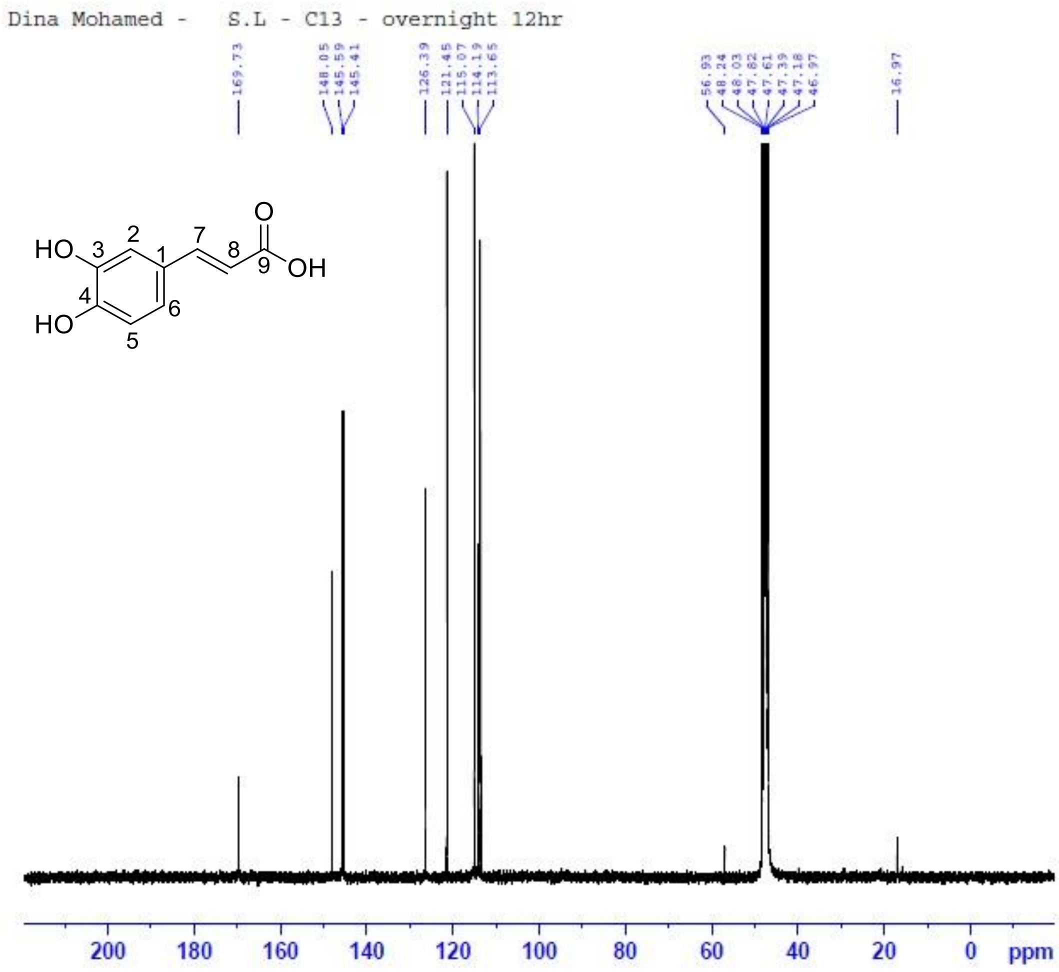

Figure S8: ¹³C-NMR spectrum of Compound 4 (125 MHz, MeOH).

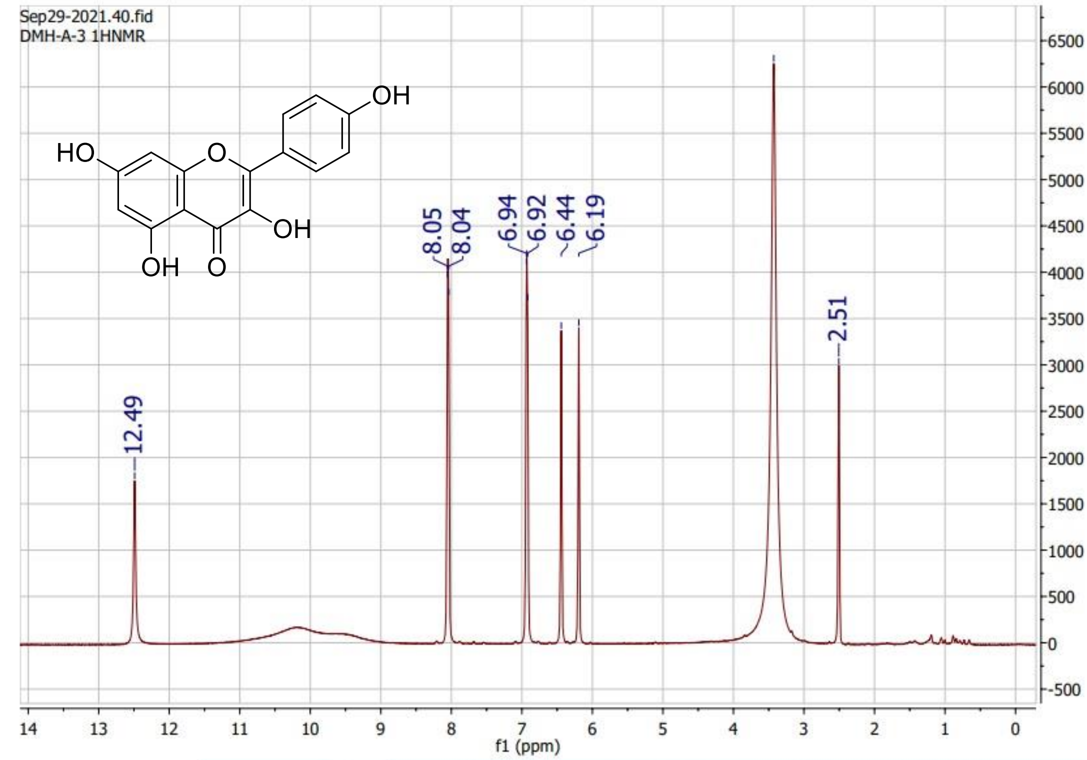

Figure S9: ^{1}H-NMR spectrum of Compound 5 (500 MHz, DMSO-d6).

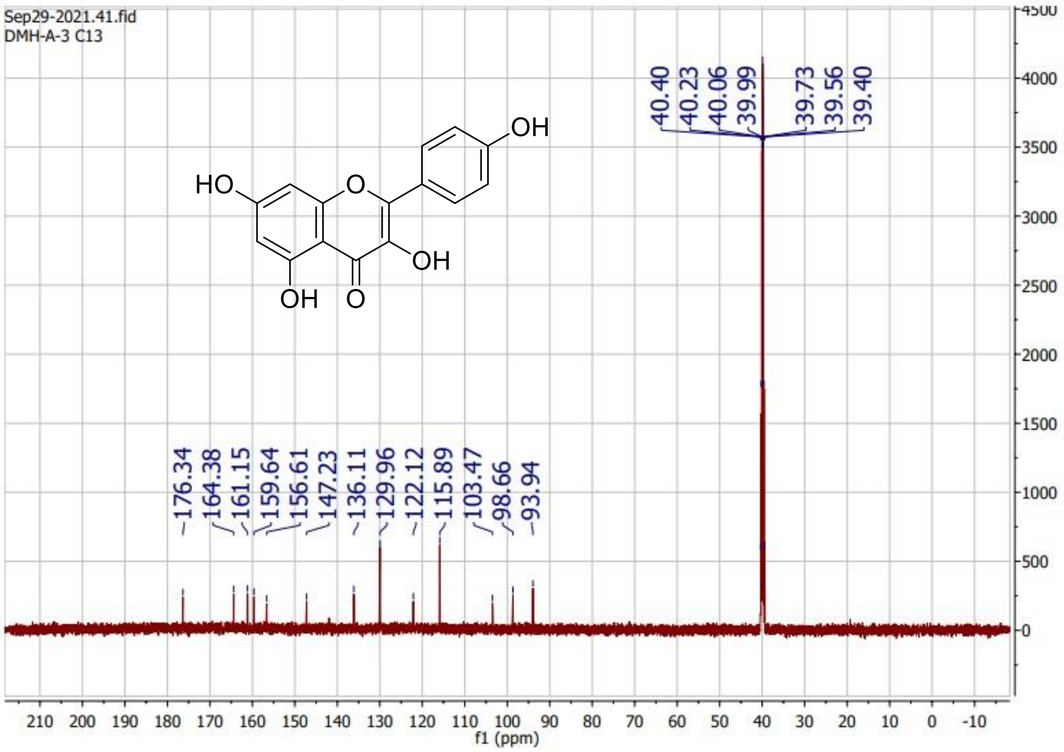

Figure S10: ^{13}C-NMR spectrum of Compound 5 (125 MHz, DMSO-d6).

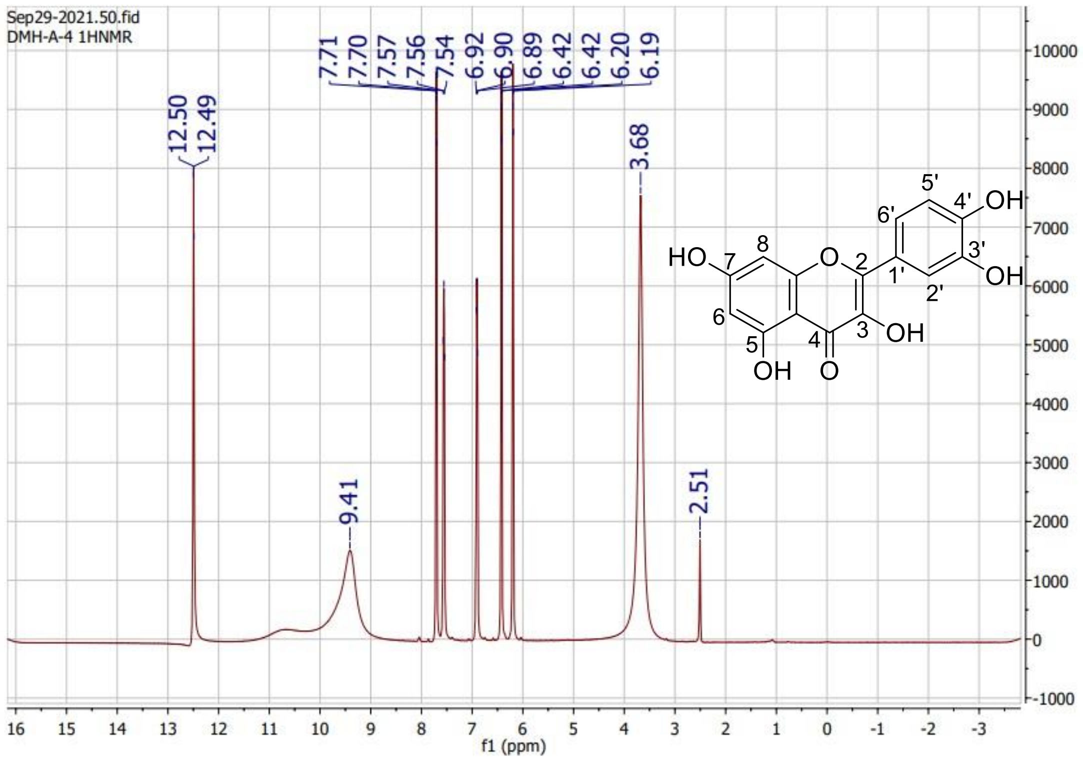

Figure S11: ^{1}H-NMR spectrum of Compound 6 (500 MHz, DMSO-d6).

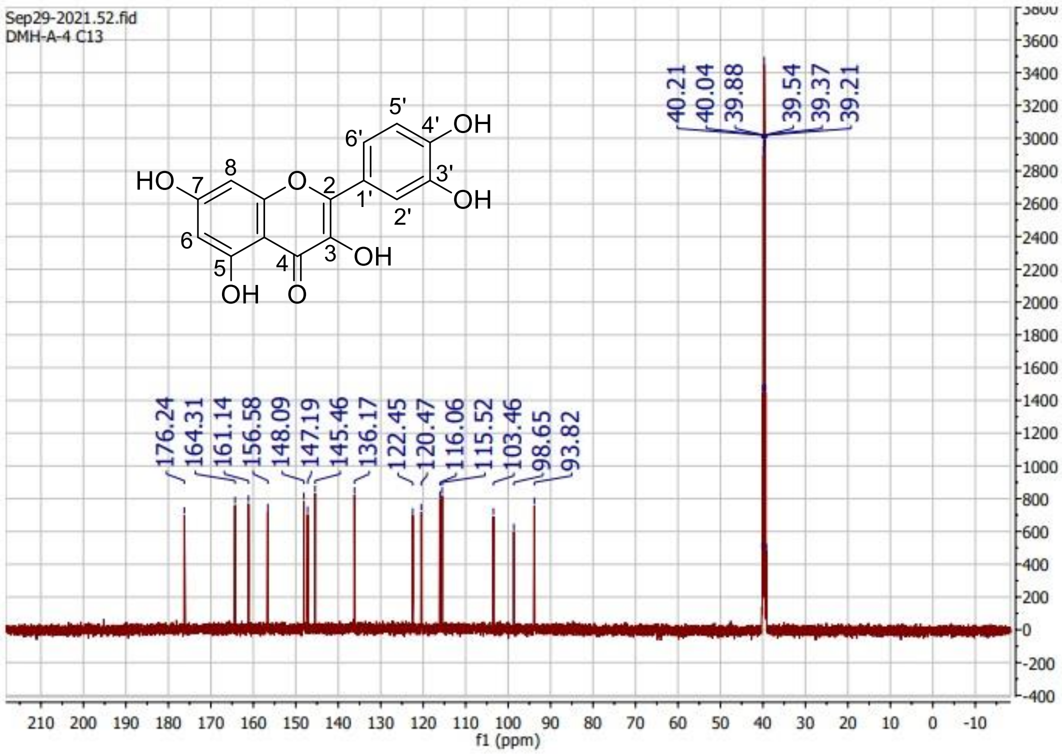

Figure S12: ^{13}C-NMR spectrum of Compound 6 (125 MHz, DMSO-d6).

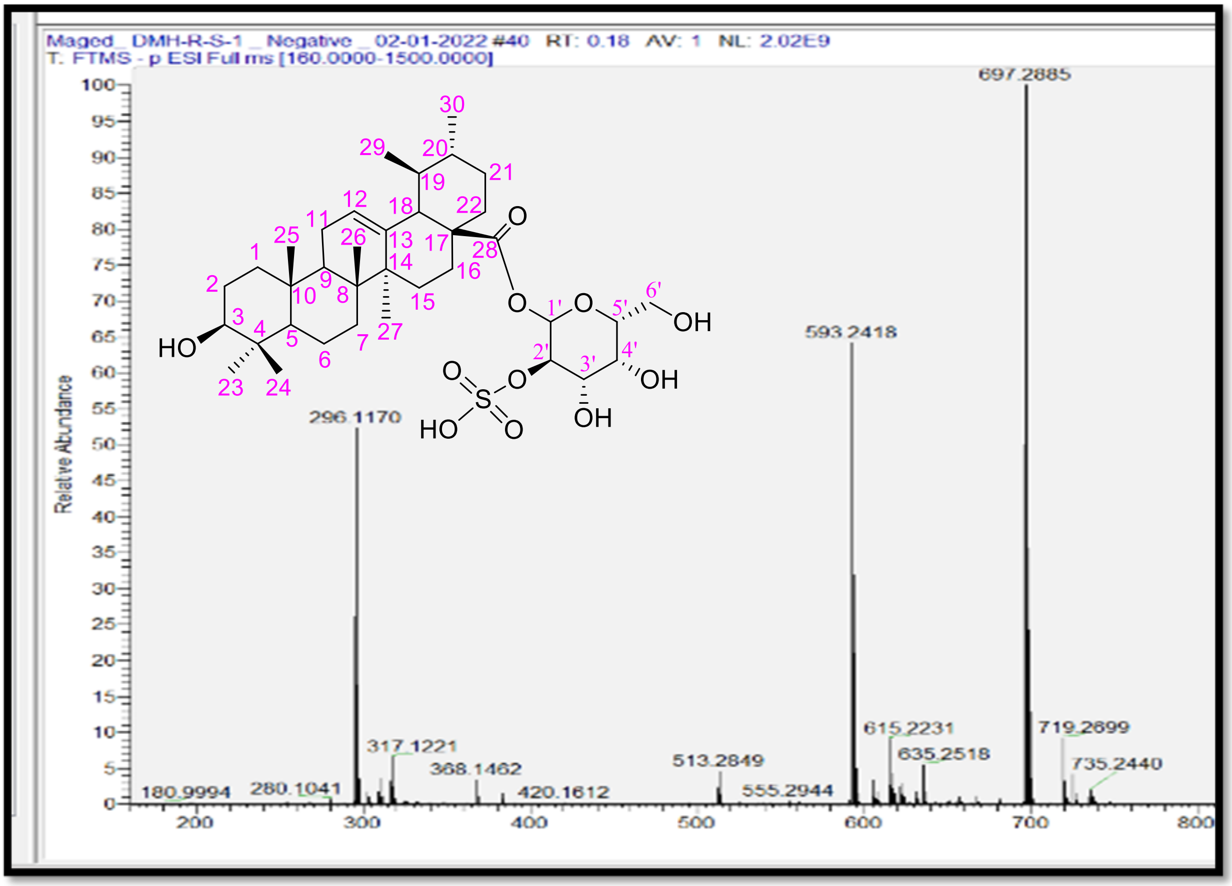

Figure S13: HRMS of Compound 7

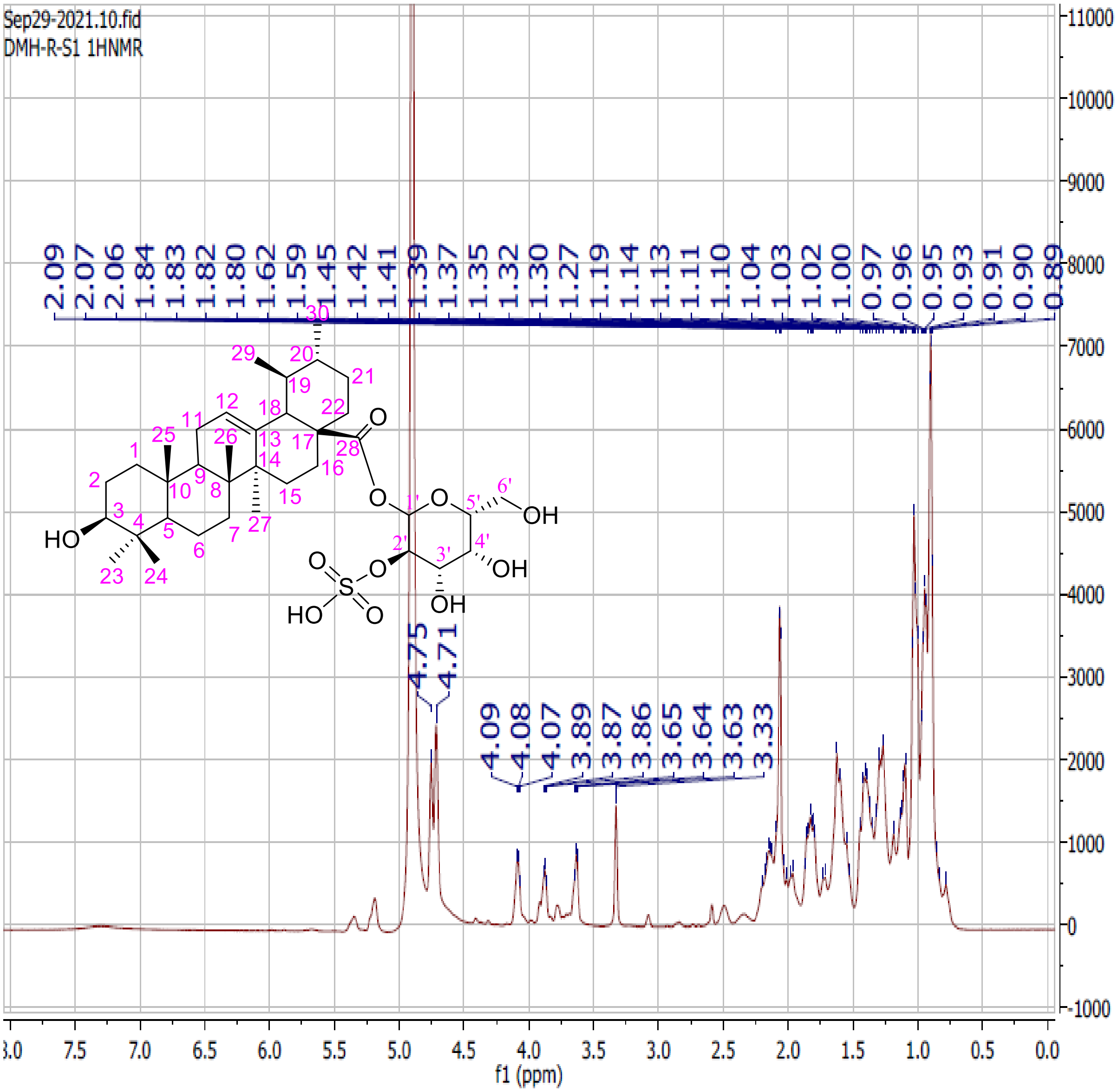

Figure S14: ^{1}H-NMR spectrum of Compound 7 (500 MHz, MeOH).

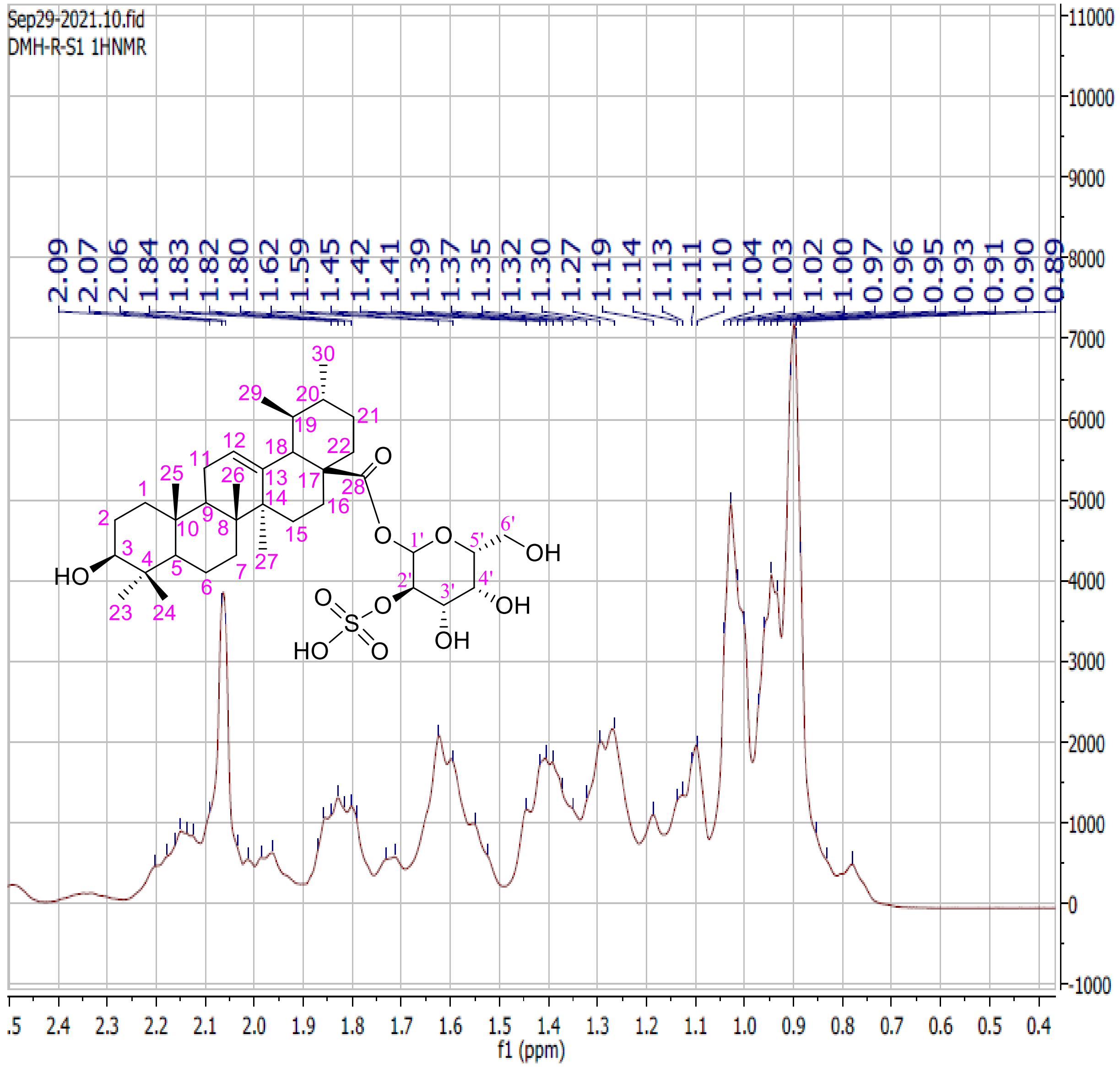

Figure S15: Partial expansion of ^{1}H-NMR spectrum of Compound 7 (500 MHz, MeOH).

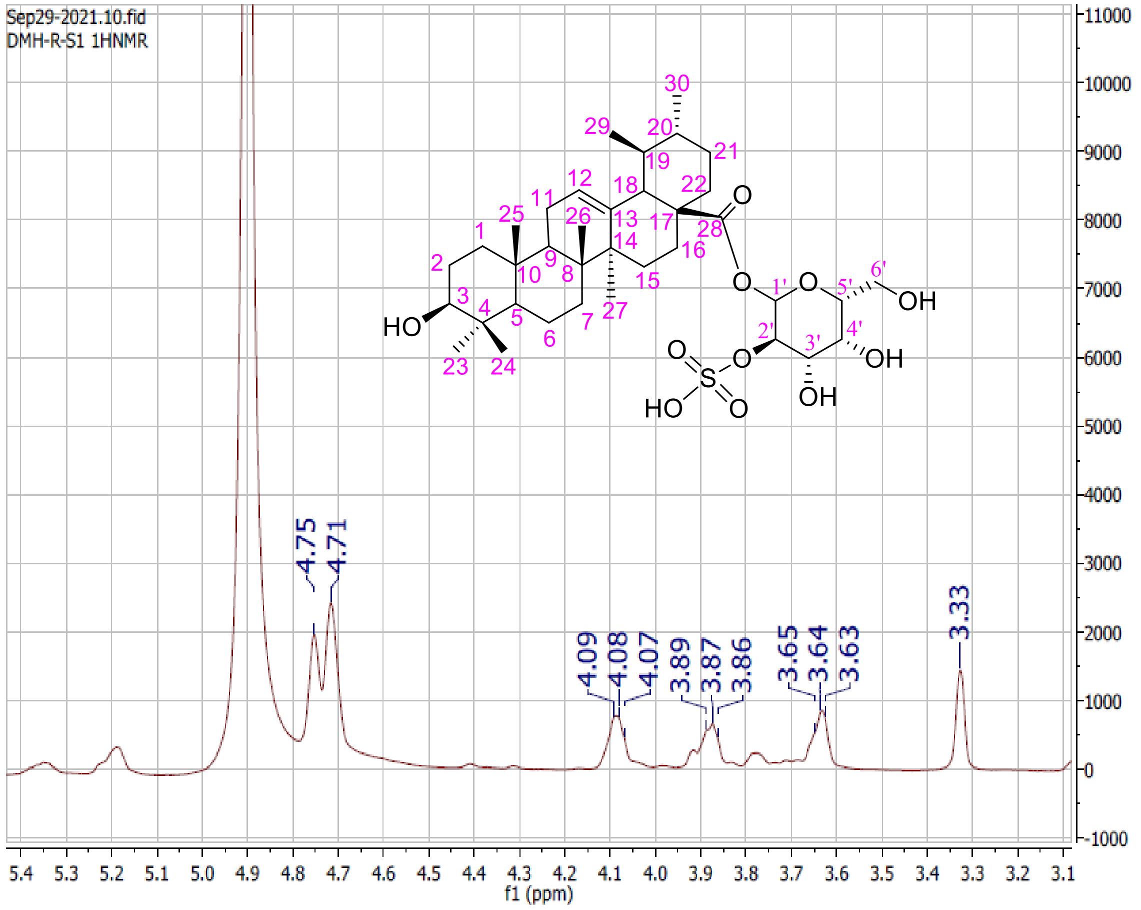

Figure S16: Partial expansion of ^{1}H-NMR spectrum of Compound 7 (500 MHz, MeOH).

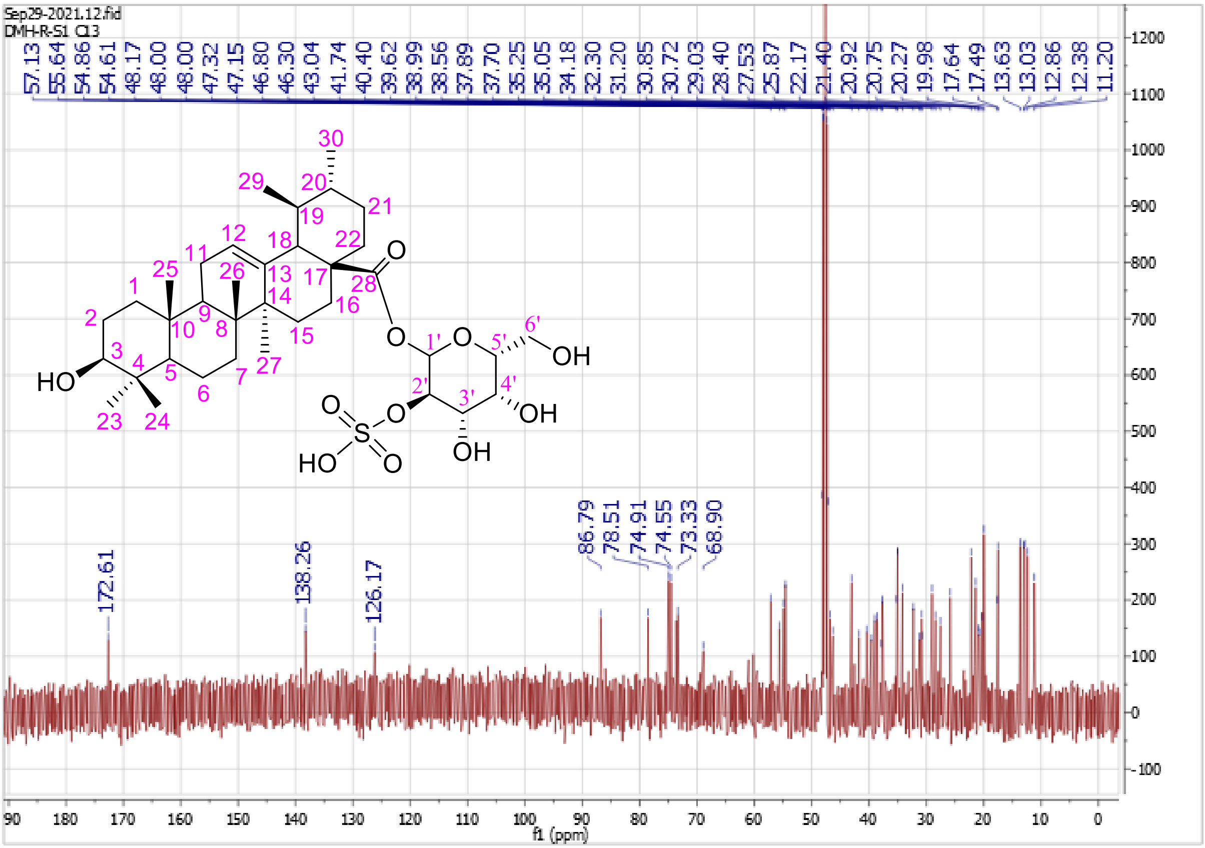

Figure S17: ^{13}C-NMR spectrum of Compound 7 (125 MHz, MeOH).

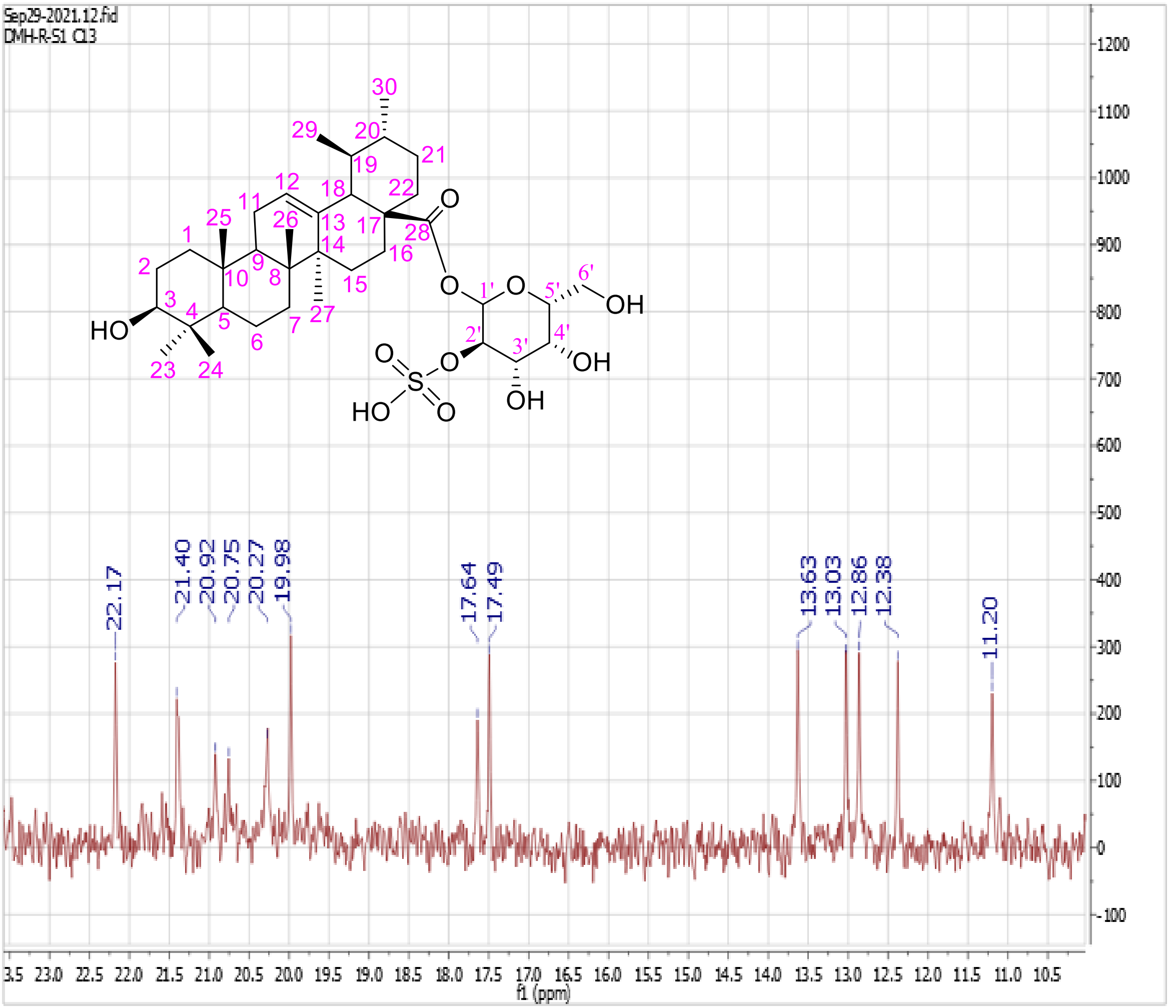

Figure S18: Partial expansion of ^{13}C-NMR spectrum of Compound 7 (125 MHz, MeOH).

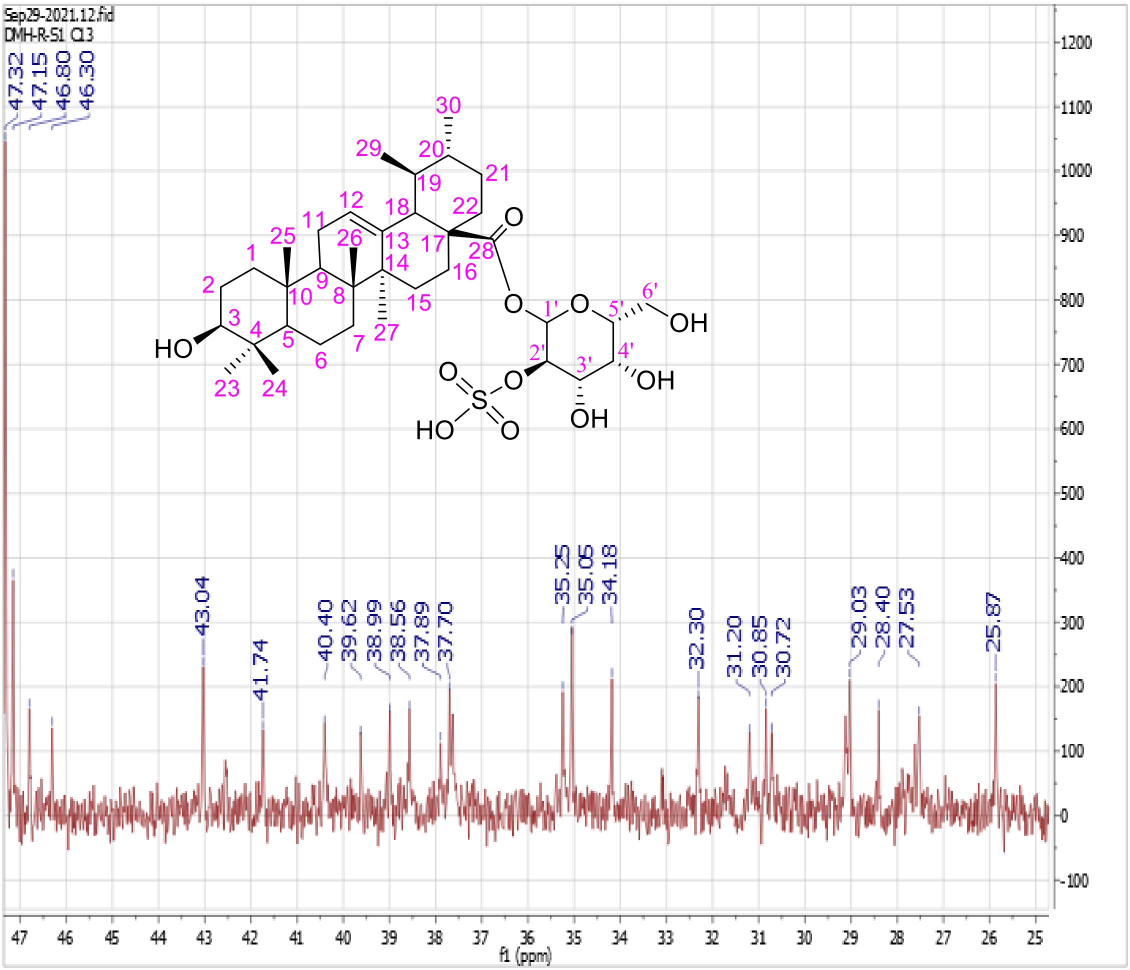

Figure S19: Partial expansion of ^{13}C-NMR spectrum of Compound 7 (125 MHz, MeOH).

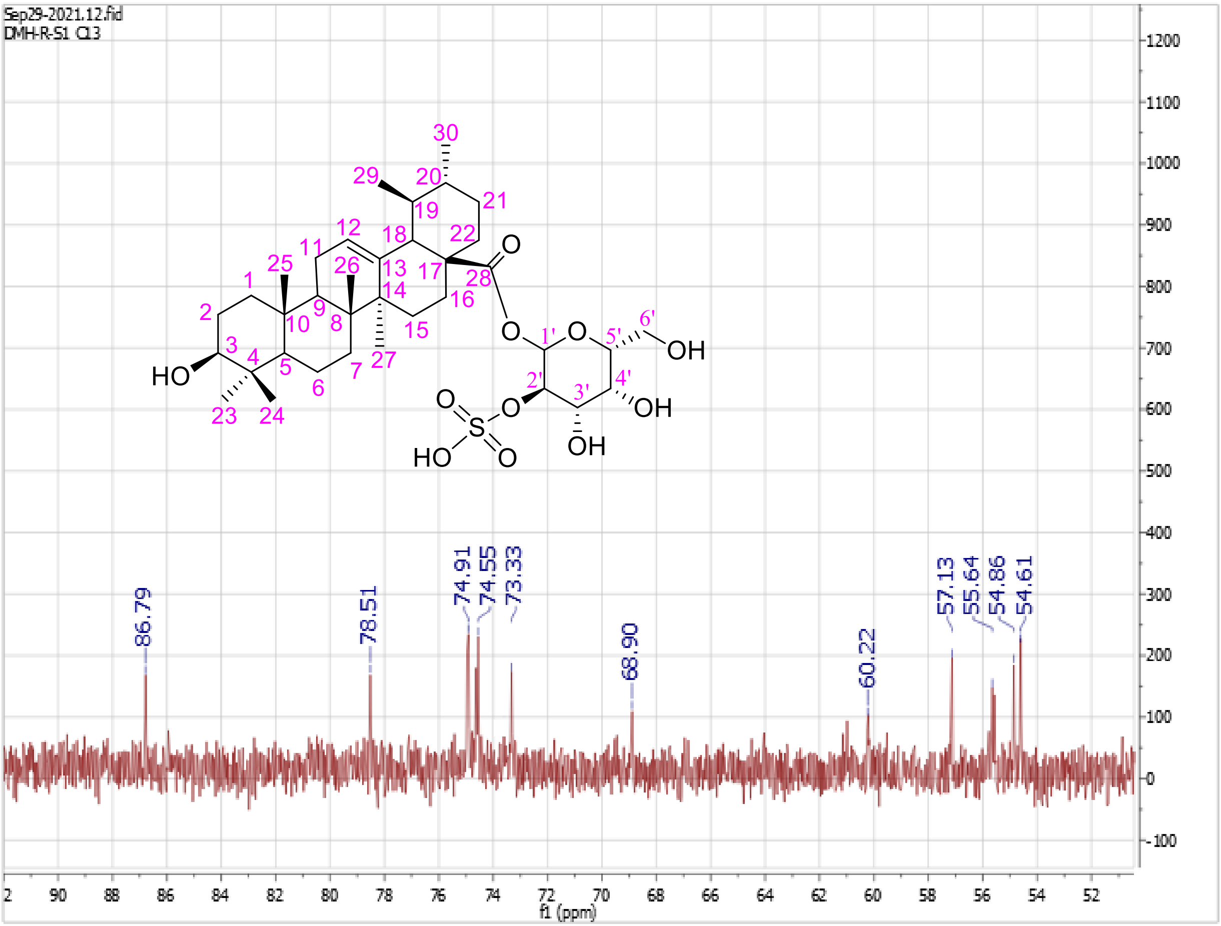

Figure S20: Partial expansion of ^{13}C-NMR spectrum of Compound 7 (125 MHz, MeOH).

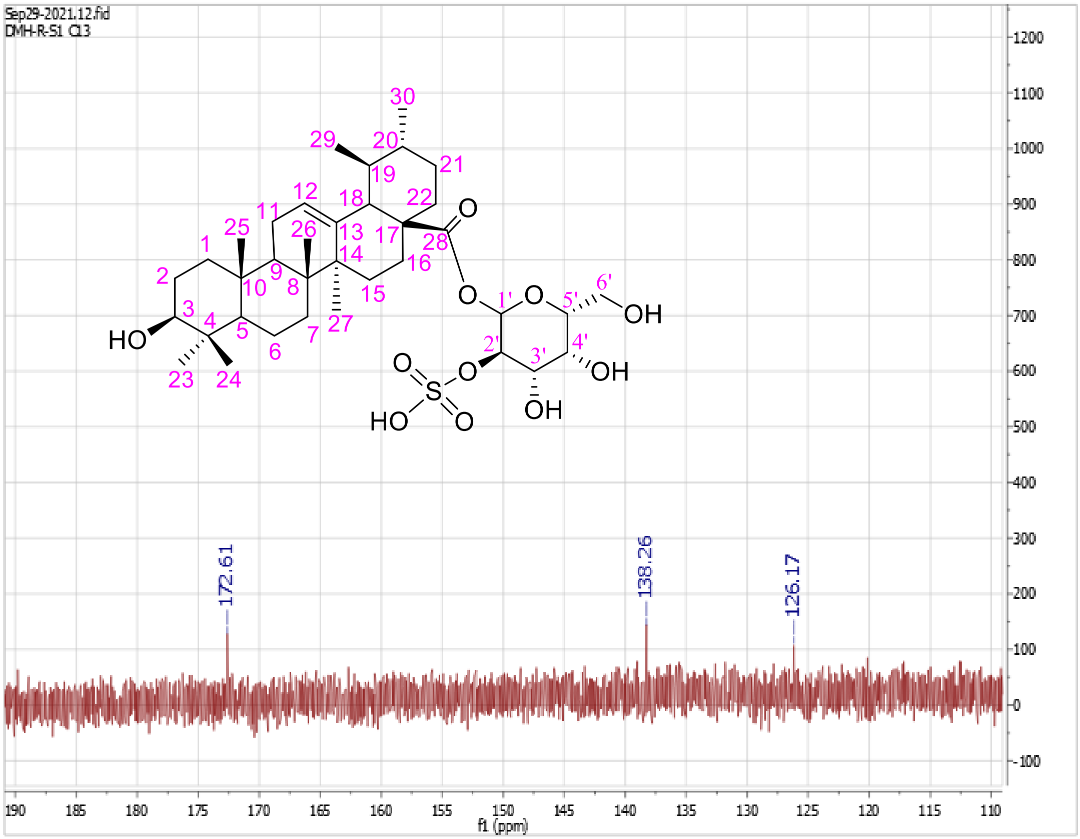

Figure S21: Partial expansion of ^{13}C-NMR spectrum of Compound 7 (125 MHz, MeOH).

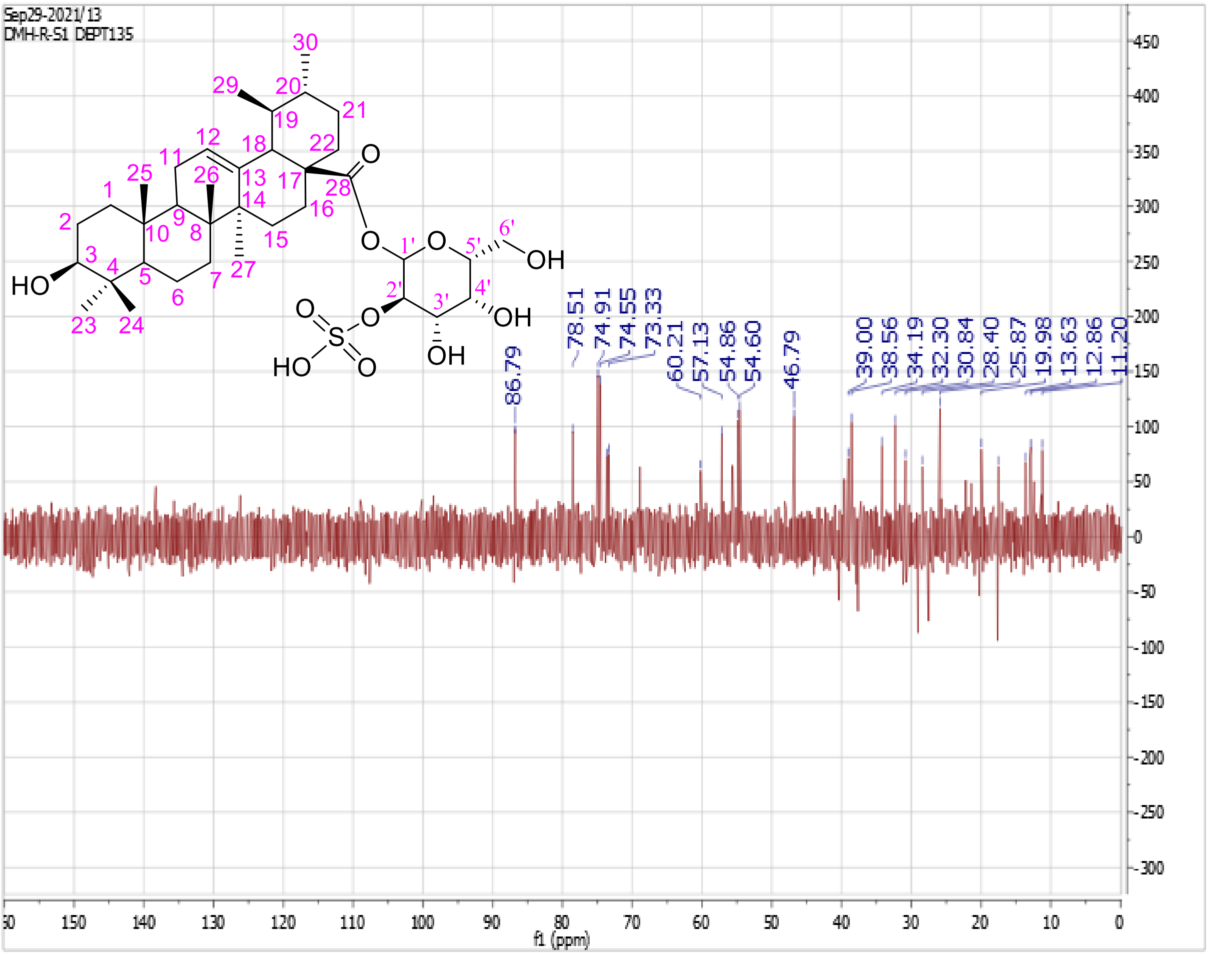

Figure S22: DEPT 135 of Compound 7 (125 MHz, MeOH).

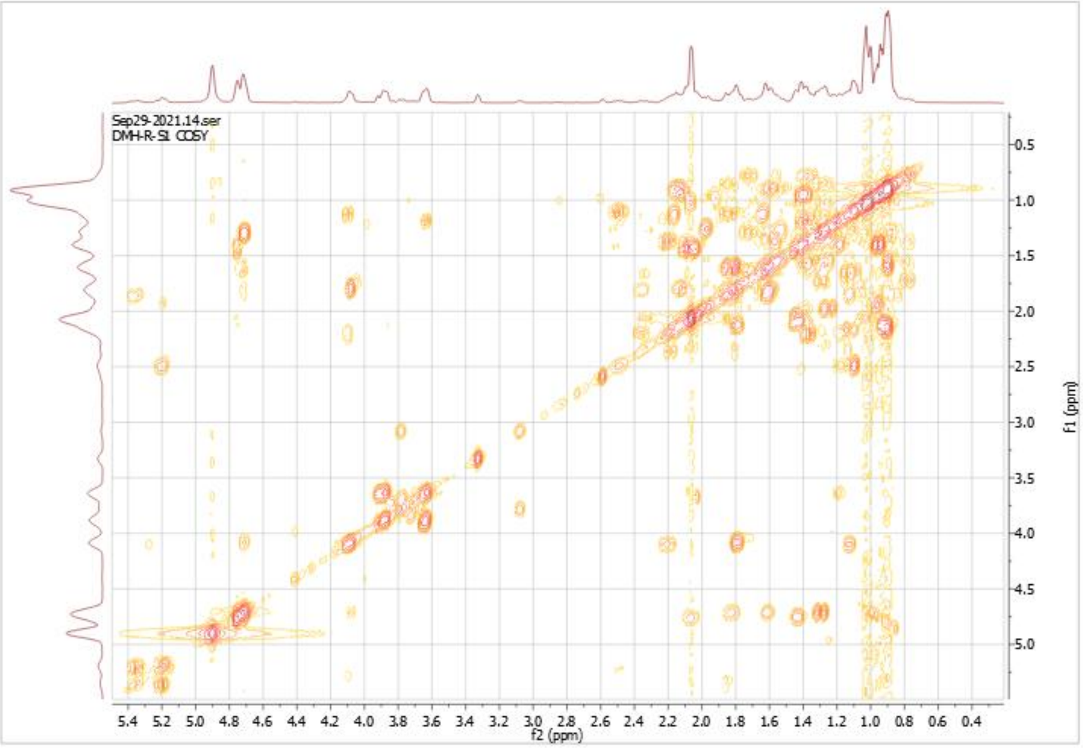

Figure S23: COSY of Compound 7

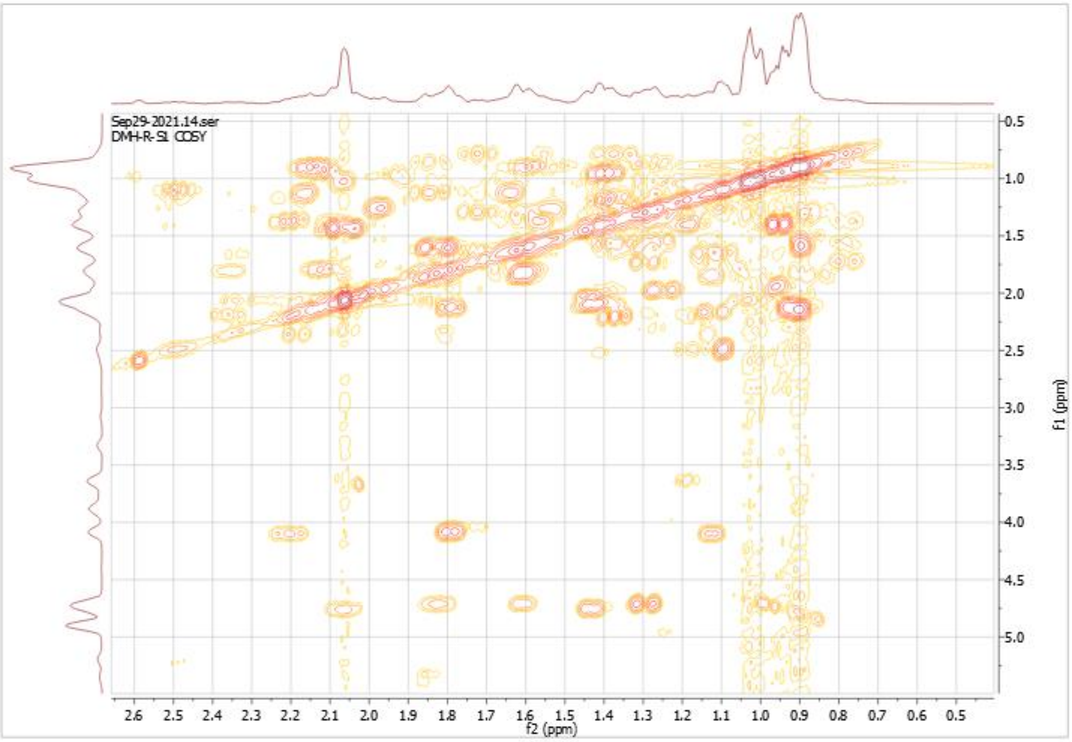

Figure S24: Partial expansion of COSY of Compound 7.

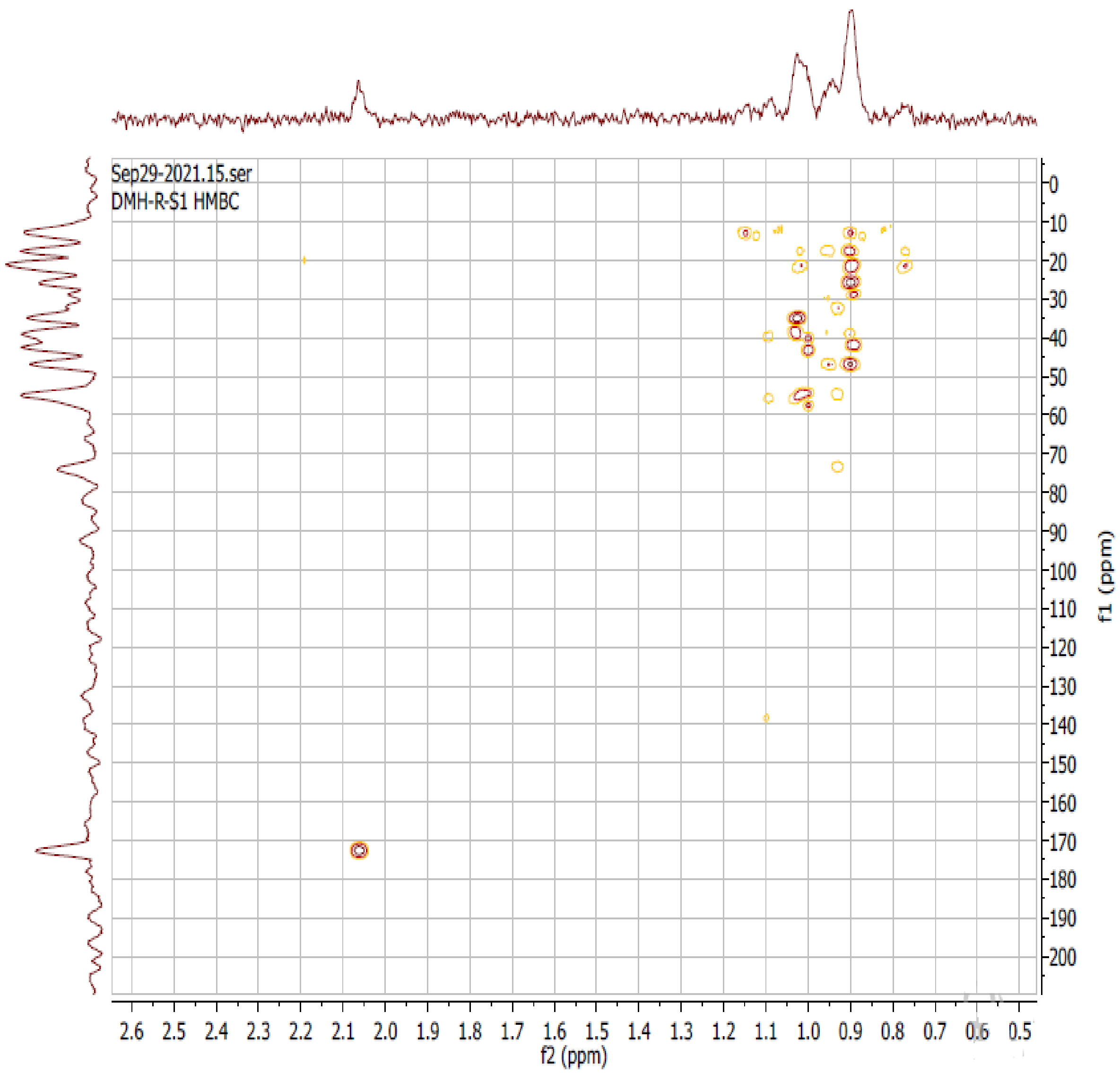

Figure S25: HMBC of compound 7

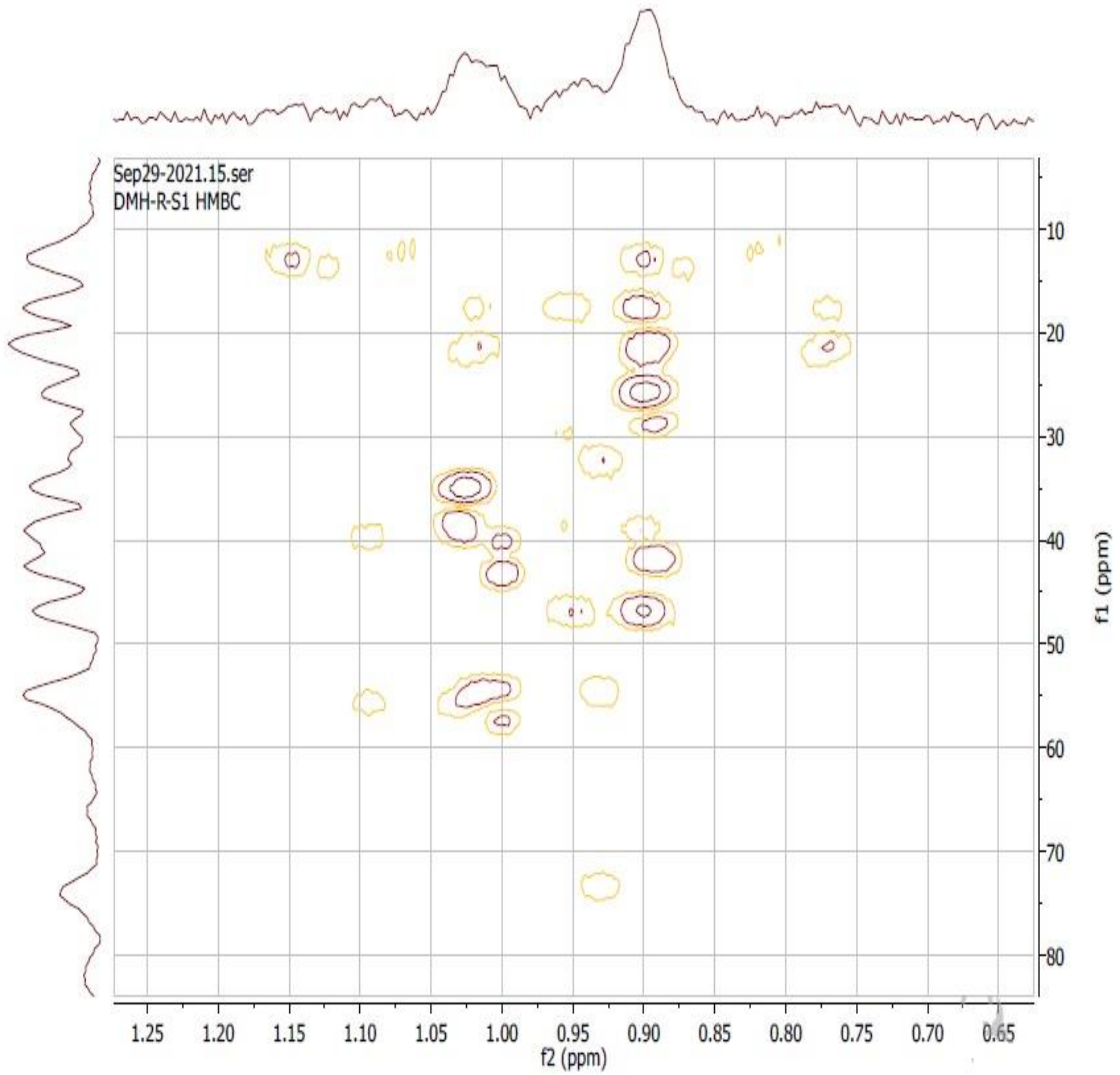

Figure S26: Expansion of HMBC of compound 7.

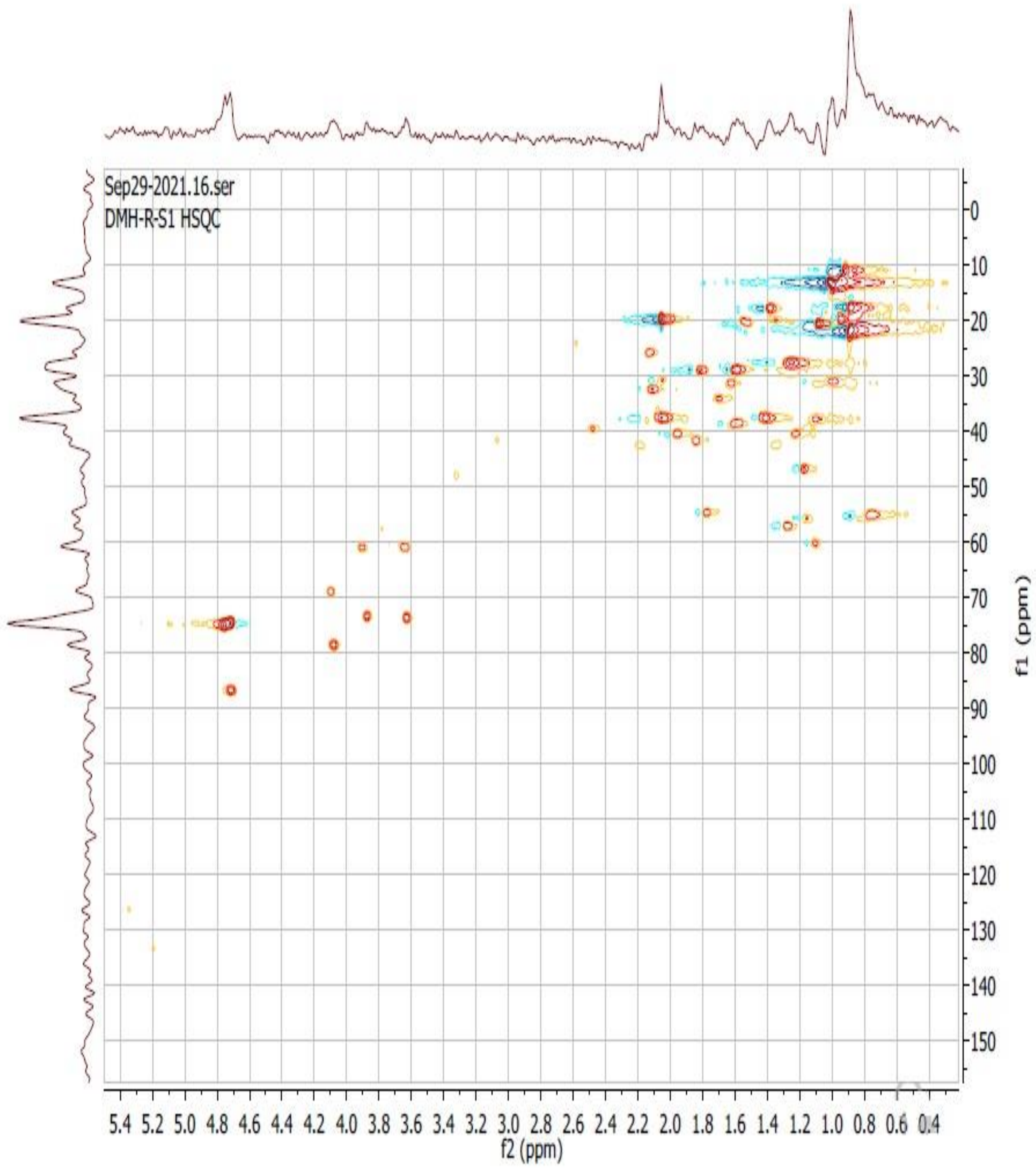

Figure S27: HSQC of compound 7

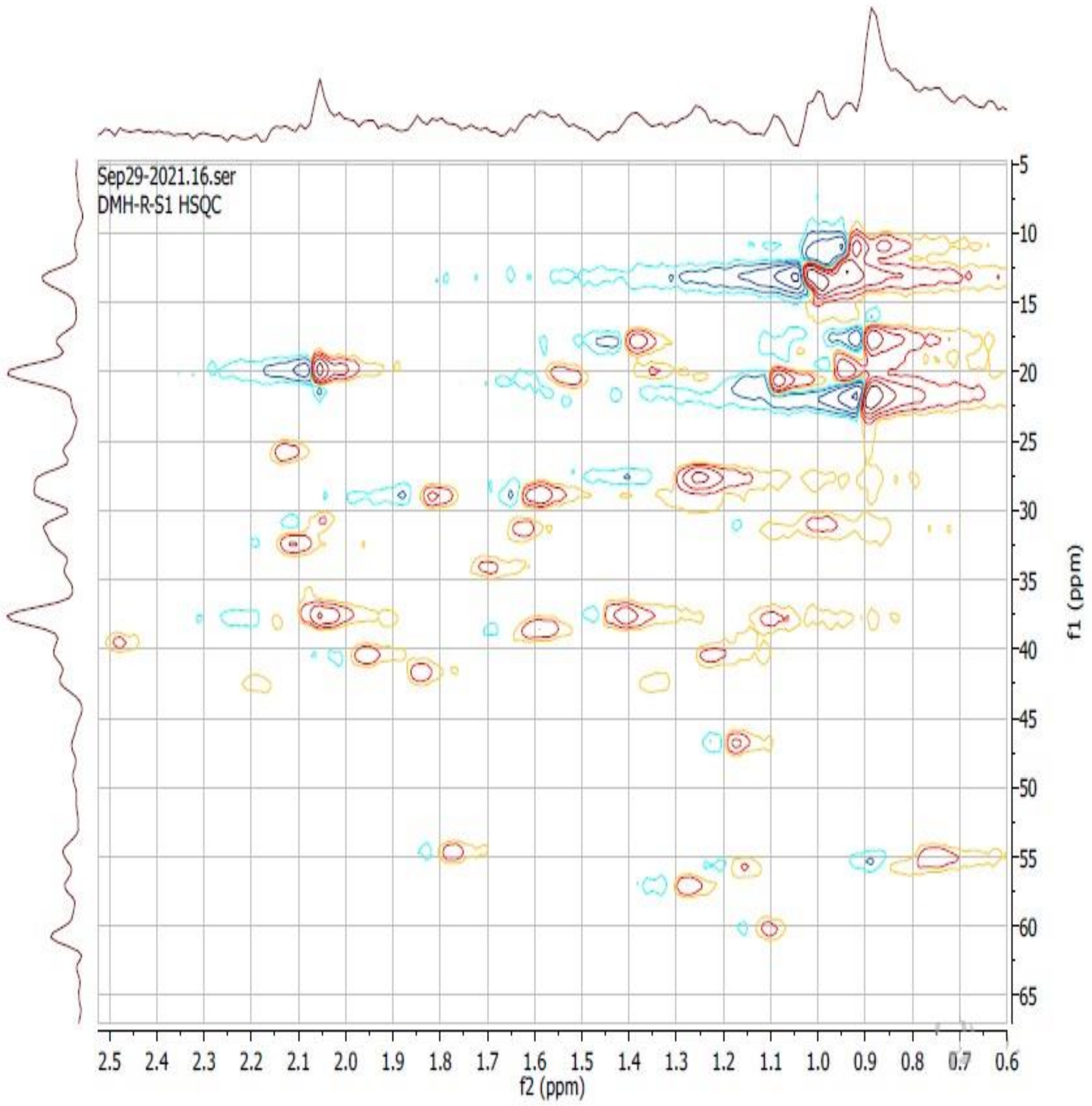

Figure S28: Expansion of HSQC of compound 7.

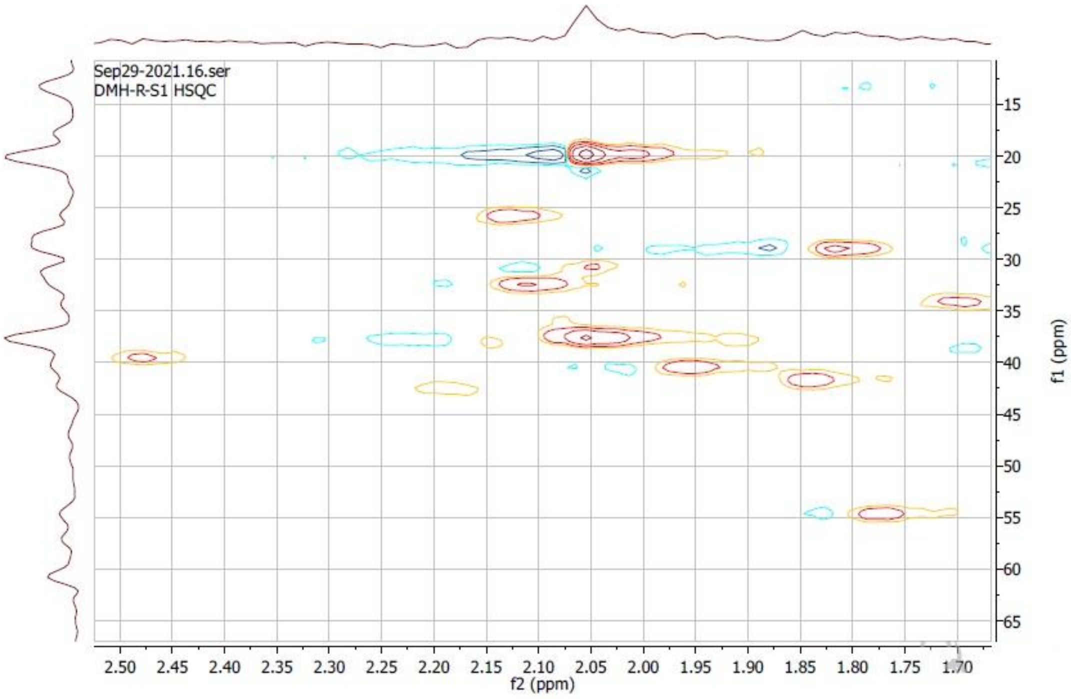

Figure S29: Expansion of HSQC of compound 7.

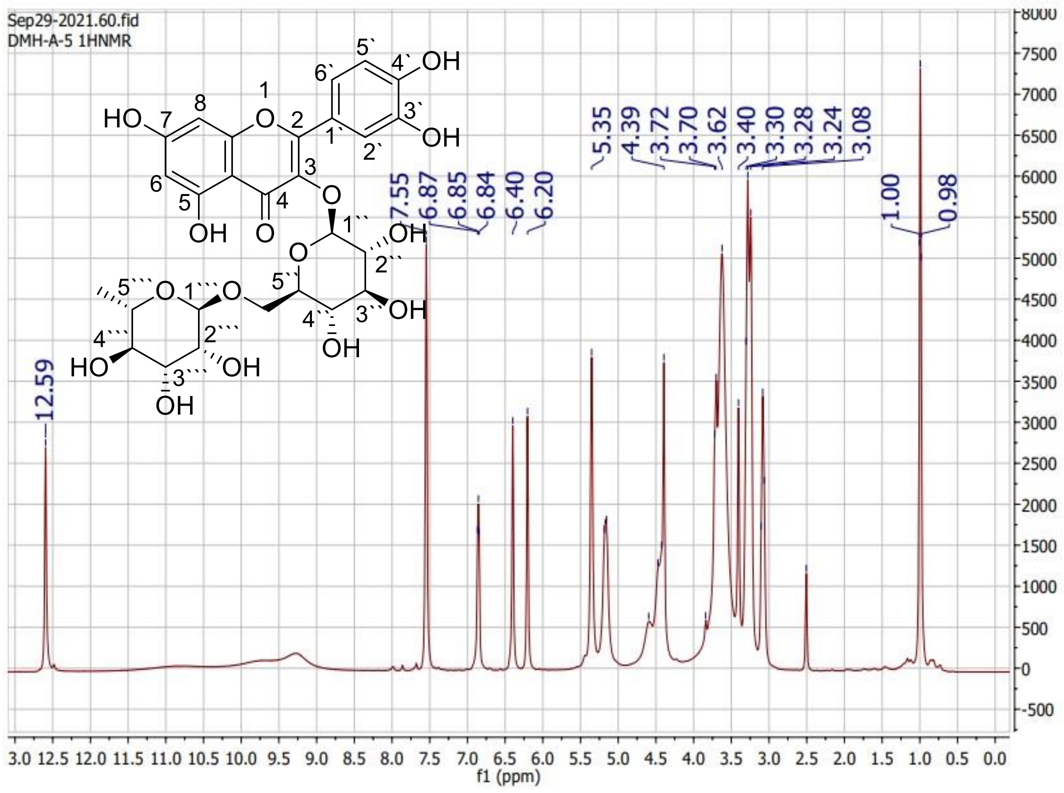

Figure S30: ^{1}H-NMR spectrum of Compound 8 (500 MHz, DMSO-d6).

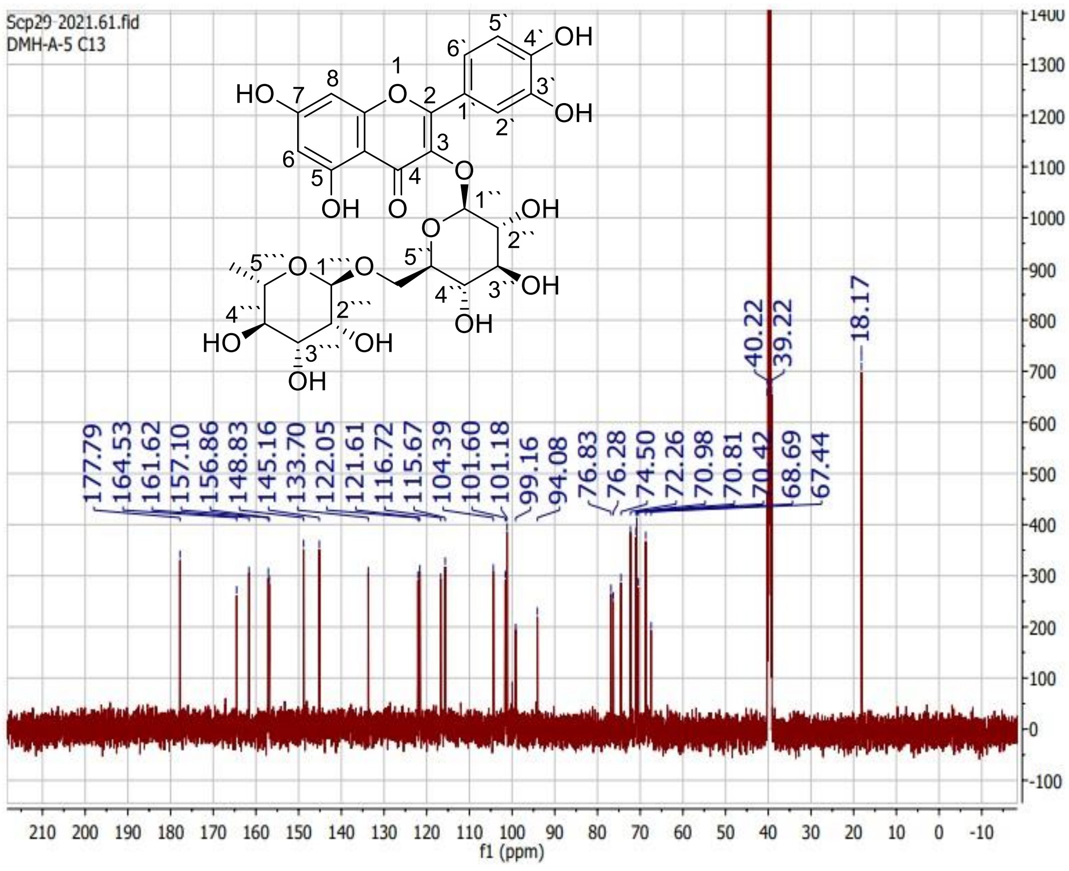

Figure 31: ^{13}C-NMR spectrum of Compound 8 (125 MHz, DMSO-d6).

Table S1. Liver enzymes and kidney markers in the groups of the preliminary study

	ALT (IU/L)	AST (IU/L)	Urea (mg/dL)	Creatinine (mg/dL)
Normal control	45.60 ± 5.00	50.79 ± 5.90	19.03 ± 3.23	0.92 ± 0.13
Z. album extract (100 mg/kg)	44.22 ± 4.11	50.36 ± 5.21	20.29 ± 3.25	0.90 ± 0.20
Zygo-albuside A (10 mg/kg)	46.32 ± 5.03	51.04 ± 4.32	20.19 ± 3.29	0.91 ± 0.18
Rutin (10 mg/kg)	45.29 ± 5.08	49.89 ± 4.90	20.21 ± 3.50	0.92 ± 0.19

Values are expressed as mean ± SD (n = 5). Data were analyzed using ANOVA followed by Tukey's post hoc test.

Table S2. Primer sequences and annealing temperatures for genes amplification.

	Forward primer	Reverse primer	Annealing temperature
NF-κb	5'-CAATGGCTACACAGGACCA-3'	5′-CACTGTCACCTGGAACCAGA-3′	52 °C
TNF-α	5'-TCTACTGAACTTCGGGGTGATCG-3'	5'-TGATCTGAGTGTGAGGGTCTGGG-3'	56 °C
p53	5'-ACCGCCGACCTATCCTTACC-3'	5'-TCTTCTGTACGGCGGTCTCTC-3'	56 °C
β-actin	5'-ACGGCCAGGTCATCACTATTG-3'	5'-CAAGAAGGAAGGCTGGAAAAGA-3'	52 °C
miR-29a	5′-GCGCACTGATTTCTTTTGGTGTTCAG–3'	5′-GCGAGCACAGAATTAATACGAC-3'	51 °C
RNU6B	5′-CTCGCTTCGGCAGCACATA–3'	5′-CGCTTCACGAATTTGCGTG-3'	53 °C
